# The Antiprotozoal Activity of Papua New Guinea Propolis and Its Triterpenes

**DOI:** 10.3390/molecules27051622

**Published:** 2022-03-01

**Authors:** Samya S. Alenezi, Naif D. Alenezi, Godwin U. Ebiloma, Manal J. Natto, Marzuq A. Ungogo, John O. Igoli, Valerie A. Ferro, Alexander I. Gray, James Fearnley, Harry P. de Koning, David G. Watson

**Affiliations:** 1 Strathclyde Institute of Pharmacy and Biomedical Science, University of Strathclyde, 161 Cathedral Street, Glasgow G4 0RE, UK; sameahq8@gmail.com (S.S.A.); ndenzi@sfda.gov.sa (N.D.A.); v.a.ferro@strath.ac.uk (V.A.F.); a.i.gray345@gmail.com (A.I.G.); 2 Institute of Infection, Immunity and Inflammation, College of Medical, Veterinary and Life Sciences, University of Glasgow, Glasgow G12 8TA, UK; g.ebiloma@tees.ac.uk (G.U.E.); manal.natto@glasgow.ac.uk (M.J.N.); 2226184u@student.gla.ac.uk (M.A.U.); j.o.igoli@uam.edu.ng (J.O.I.); 3 School of Health and Life Sciences, Teesside University, Middlesbrough TS1 3BX, UK; 4 Phytochemistry Research Group, Department of Chemistry, University of Agriculture, Makurdi PMB 2373, Nigeria; 5 BeeVital, Whitby, North Yorkshire YO22 5JR, UK; james.fearnley@beevitalpropolis.com

**Keywords:** triterpenes, *T. brucei*, *T. congolense*, Papua New Guinea, propolis, U947 cells

## Abstract

Profiling a propolis sample from Papua New Guinea (PNG) using high-resolution mass spectrometry indicated that it contained several triterpenoids. Further fractionation by column chromatography and medium-pressure liquid chromatography (MPLC) followed by nuclear magnetic resonance spectroscopy (NMR) identified 12 triterpenoids. Five of these were obtained pure and the others as mixtures of two or three compounds. The compounds identified were: mangiferonic acid, ambonic acid, isomangiferolic acid, ambolic acid, 27-hydroxyisomangiferolic acid, cycloartenol, cycloeucalenol, 24-methylenecycloartenol, 20-hydroxybetulin, betulin, betulinic acid and madecassic acid. The fractions from the propolis and the purified compounds were tested in vitro against *Crithidia fasciculata*, *Trypanosoma congolense*, drug-resistant *Trypanosoma congolense, Trypanosoma b. brucei* and multidrug-resistant *Trypanosoma b. brucei* (B48). They were also assayed for their toxicity against U947 cells. The compounds and fractions displayed moderate to high activity against parasitic protozoa but only low cytotoxicity against the mammalian cells. The most active isolated compound, 20-hydroxybetulin, was found to be trypanostatic when different concentrations were tested against *T. b. brucei* growth.

## 1. Introduction

Bees gather propolis from the buds, exudates and barks of a variety of plants, leading to wide chemical diversity. Since propolis contains a multitude of chemical components in a complex mixture, its biological activity and pharmacological properties might similarly be broad [1,2]. Propolis has been observed to have promising activity against a number of protozoal species, particularly *Trypanosoma* and *Leishmania* species [3,4,5,6,7,8,9]. Studies of the antiprotozoal activity of propolis have recently been reviewed [10]. Diseases caused by parasitic protozoa remain a problem worldwide and these diseases include human African trypanosomiasis (HAT) and animal African trypanosomiasis (AAT), which occur in Africa, and Chagas disease; these are all caused by *Trypanosoma* species. In addition, the closely related *Leishmania* parasites cause a variety of diseases throughout the world [11,12]. Chemotherapy is still important for the control of most parasitic diseases, such as trypanosomiasis and leishmaniasis, as no vaccines are available. The current treatment of human African trypanosomiasis (HAT) or animal African trypanosomiasis (AAT) is based on a few drugs which were developed decades ago. However, the current frontline drugs are quite toxic and in most cases require intravenous administration. Furthermore, resistance to current drugs by trypanosomes is another threat to effective chemotherapy [12,13].

Thus, there is a need for new treatment approaches; the progress in discovering new and effective anti-parasitic drugs has been very poor [12]. Propolis seems to be a good lead candidate as a starting point for drug discovery in view of the fact that it has been frequently found to be toxic to protozoa [10].

In the current paper, we report on the chemical profiling and antiprotozoal assay of a propolis sample from Papua New Guinea (PNG), which as far as we know has not been investigated before. We focused on activity against trypanosomatids, which cause fatal disease in humans and other animals as well as insects. *Trypanosoma* species are the most common organisms within the Trypanosomatidae class that has many different subspecies, including *Trypanosoma b. gambiense* and *Trypanosoma b. rhodesiense*, which are transmitted by the tsetse fly and cause HAT, also known as African sleeping sickness. Other trypanosome species cause AAT, including *T. b. brucei*, *T. congolense* and *T. vivax* [14]. The genus *Crithidia* contains a number of species with a wide host range, depending upon the species of the parasite. *C. fasciculata* has been widely used as a model organism in research of trypanosomatid biology that may then be applied to understanding the biology of the human infective species [15]. *Crithidia* species parasitise several species of insects including bees and have been reported as a possible cause of winter colony collapse in Europe [16]. Activity against *C. fasciculata* was also tested in order to assess the importance of propolis for preventing these types of protozoal infections in bees.

## 2. Results

### 2.1. LC-MS Profiling of the Crude PNG Propolis Sample

Approximately 38.4 g of raw PNG propolis was extracted with ethanol to obtain a crude extract weighing 29.2 g. Profiling of the PNG propolis sample by high-resolution LC-MS indicated a clear set of molecular formulae (Table 1) consistent with a series of triterpenes with various degrees of oxygenation, including some highly oxygenated compounds such as C_29_H_48_O_6_ and C_30_H_48_O_6_ and some with a high degree of unsaturation such as C_30_H_46_O_4_. The most abundant compounds from the LC-MS data were C_30_H_48_O_4_ and C_30_H_50_O_3_. To date, there have been no data published on PNG propolis in the literature with which this sample could be compared. Generally, these triterpenes appear to be different from the ones isolated from some PNG plant species such as *Terminalia* spp., in which trihydroxylated triterpene acids such as arjunolic and asiatic acids were found [17].

### 2.2. Fractionation of the PNG Extract Using Open Column Chromatography (OCC)

A portion of the crude PNG extract (3 g) was fractionated using OCC to obtain the fractions PNG F1 to PNG F10. The masses of the different fractions collected are presented in Table 2. The two fractions with the greatest weights (PNG-F1 and PNG-F6) were chosen for further separation by medium-pressure liquid chromatography (MPLC)**.**

### 2.3. In Vitro Testing of Compounds and Fractions Derived from PNG Propolis against T. b. brucei, T. congolense and C. fasciculata

Alamar blue (resazurin) assays were used to obtain the EC_50_ values for PNG and its fractions in at least three independent determinations. Table 3 shows the results obtained from the assay for antitrypanosomal effects of the PNG sample and its fractions, using wild-type *T. b. brucei* s427 and the derived multidrug-resistant cell line *T. b. brucei* B48. The most active fractions were fractions F4, F5 and F6. A notable observation was that none of the fractions and purified compounds showed reduced activity against the drug-resistant strain B48 (i.e., RF < 1 and therefore not cross-resistant with first-line trypanosomiasis drugs such as dimamidines, including pentamidine, used in these assays as control, or melaminophenyl arsenicals) [18,19,20]. Indeed, the trypanocidal activity of most PNG fractions was significantly higher (*p* < 0.05) against B48, often around two-fold. In contrast, the resistance factor (RF) for pentamidine was 210.8, with a *p* value of 0.00001.

### 2.4. Testing of the Cytotoxicity of PNG Extract and Its Fractions against U937 Cells

The cytotoxicity of the crude extract and ten fractions was assessed against U937 cells. The results show that toxicity of PNG propolis was low (Table 4), as is generally observed for propolis extracts [3,4,5,6].

### 2.5. Characterisation of MPLC Subfractions Derived from OCC Fractions

Figure S1 shows workflows for the extraction and fractionation of the PNG propolis sample. In addition, PNG-F4 was selected for further fractionation in view of its high activity against *T. brucei* (Table 3) and good weight of material; it was separated into subfractions by a repeat of OCC.

#### 2.5.1. Identification of MPLC Fraction PNG-F1-5 as a Mixture of Cycloartenol, 24 (28)-Methylenecycloartenol and Cycloeucalenol

Gas chromatography-mass spectrometry (GC-MS) analysis of PNG-F1-5B indicated a mixture of three components (Figure S2). The high-resolution LC-MS analysis of PNG-F1-5B showed three peaks: at 27.4 min having an [M + H]^−^ ion at *m*/*z* 425.3426 with an elemental composition C_30_H_49_O; at 30.4 min having an [M + H]^−^ ion at *m*/*z* 439.3034 with an elemental composition C_31_H_51_O; and at 34.0 min having an [M + H]^−^ ion at *m*/*z* 425.3424 with an elemental composition C_30_H_49_O. The three components in the mixture were identified as cycloartenol (Figure S3), 28-methylenecycloartenol (Figure S4) and cycloeucalenol (Figure S5). Details of the NMR results are given in Figures S6–S9. The chemical shifts for the compounds (Table S1) were confirmed using literature reports [21,22].

#### 2.5.2. Characterisation of PNG-F4-11 as Betulin

The compound had an [M + H]^+^ ion at *m/z* 443.3884, corresponding with the molecular formula C_30_H_51_O_2_. The NMR data are shown in Table S2 and the structure of betulin (Figure S10) was confirmed by comparison with the literature [23]. The ^1^H NMR and ^13^C NMR spectra are shown in Figures S11 and S12.

#### 2.5.3. Characterisation of PNG-F4-13 as Betulinic Acid

The compound had an [M + H]^+^ ion at *m/z* 457.3675, corresponding with the molecular formula C_30_H_49_O_3_. The NMR data (Table S3) for betulinic acid (Figure S13) were confirmed by comparison with the literature [24,25,26]. The ^1^H NMR and ^13^C NMR spectra are shown in Figures S14 and S15.

#### 2.5.4. Characterisation of PNG-F4-18 as Madecassic Acid

The compound had an [M − H]^−^ ion at *m*/*z* 503.3378 corresponding to the molecular formula C_30_H_47_O_6_ (Figure S16). The NMR data (Table S4) for madecassic acid were confirmed by comparison with the literature [24,25,26]. The ^1^H NMR and ^13^C NMR spectra are shown in Figures S17 and S18.

#### 2.5.5. Identification of F5 as 20-Hydroxybetulin

The compound had an [M − H]^−^ ion at *m*/*z* 459.3850 corresponding to the molecular formula C_30_H_51_O_3_. Comparison of the NMR data (Table S5) with the literature [23] confirmed the structure as 20-hydroxybetulin (Figure S19). The ^1^H NMR and ^13^C NMR spectra are shown in Figures S20 and S21.

#### 2.5.6. Characterisation of PNG-F6-S12 as a Mixture of Mangiferonic Acid and Ambonic Acid

High-resolution LC-MS analysis of PNG-F6-12 showed two peaks at 25.1 min having an [M + H]^+^ ion at *m*/*z* 455.3508 with elemental composition C_30_H_47_O_3_, and at 28.6 min having an [M + H]^+^ ion at *m*/*z* 469.3666 with elemental composition C_31_H_49_O_3_. The ^1^H NMR and ^13^C NMR spectra (Table S6) were compared with the literature [27,28] and were consistent with the sample being a mixture of mangiferonic (Figure S22) and ambonic acid (Figure S23). The ^1^H NMR and ^13^C NMR spectra are shown in Figures S24 and S25.

#### 2.5.7. Characterisation of PNG-F6-S15 as a Mixture of Isomangiferolic Acid and Ambolic Acid

High-resolution LC-MS analysis of PNG-F6-15 showed two peaks at 23.0 min having an [M + H]^−^ ion at *m*/*z* 469.3681 with elemental composition C_31_H_49_O_3_, and at 30.0 min having an [M + H]^−^ ion at *m*/*z* 455.3526 with elemental composition C_30_H_47_O_3_. The ^1^H NMR and ^13^C NMR spectra (Table S7) were compared with the literature [27,28] and were consistent with the sample being a mixture of mangiferonic (Figure S26) and ambolic acid (Figure S27). The ^1^H NMR and ^13^C NMR spectra are shown in Figures S28 and S29.

#### 2.5.8. Characterisation of PNG-S17 as 27-Hydroxyisomangiferolic Acid

High-resolution LC-MS analysis of PNG-S17 showed a peak at 26.6 min (Figure S28) having an [M − H]^−^ ion at *m*/*z* 471.3475 with elemental composition C_30_H_47_O_4_. The ^1^H NMR and ^13^C NMR spectra (Table S8) were compared with the literature [27,28] and were consistent with the sample being 27-hydroxyisomangiferolic acid (Figure S30). The ^1^H NMR and ^13^C NMR spectra are shown in Figures S31 and S32.

### 2.6. In Vitro Testing of Compounds of PNG Propolis against T. b. brucei, T. congolense and C. fasciculata

The purified compounds/mixtures derived from PNG propolis were tested against wild-type *T. b. brucei* s427 and the derived multidrug-resistant cell line *T. b. brucei* B48 (Table 5). Of the purified compounds, betulin and hydroxybetulin showed significantly higher activity against B48, and hydroxybetulin was by far the most active compound against *T. b. brucei*, with an EC_50_ of just 2.0 ± 0.1 µg/mL.

#### 2.6.1. Testing of Compounds and Fractions Derived from PNG Propolis against a Drug-Sensitive (IL3000) and a Diminazene-Resistant Strain (6C3) of *T. congolense*

In much of sub-Saharan Africa, trypanosomiasis in (domesticated) animals is caused primarily by infection with *T. congolense*; the condition is treated predominantly with diminazene aceturate but resistance to this drug is widespread [11]. Both the primary fractions of PNG propolis and the isolated compounds were tested on a standard drug-sensitive strain of *T. congolense*, IL3000, and the derived diminazene-resistant cell line 6C3 [29]. The crude extract displayed promising activity against *T. congolense* (Table 6 and Table 7), including the resistant line, very close to the value obtained against *T. b. brucei* (Table 3), but none of the individual fractions matched this. Of the isolated compounds, hydroxybetulin again displayed the most potent activity, at 5.8 ± 1.3 µg/mL, and was, as for *T. b. brucei*, significantly more active against the resistant strain (*p* = 0.017).

#### 2.6.2. Testing of PNG Fractions and Isolated Compounds against U937 Cells

The purified compounds were tested for their effects on human cell line U937 and to determine whether the antiprotozoal activity is the result of general toxicity or is more specifically antiprotozoal. The toxicity of the purified compounds and fractions was found to be low against the mammalian cells (Table 8).

#### 2.6.3. Activity of PNG Propolis and Its Fractions against *C. fasciculata*

The OCC fractions derived from PNG propolis were tested against *C. fasciculata* (Table 9). Most of the fractions were more potent against *Trypanosoma* spp. than against *C. fasciculata*. All the fractions exhibited similar EC_50_ values against *C. fasciculata* (20 < EC_50_ < 54). As such, the purified compounds were not retested against this species.

#### 2.6.4. The Effect of 20-Hydroxybetulin on In Vitro Growth of *T. b. brucei*

**Figure 1:**
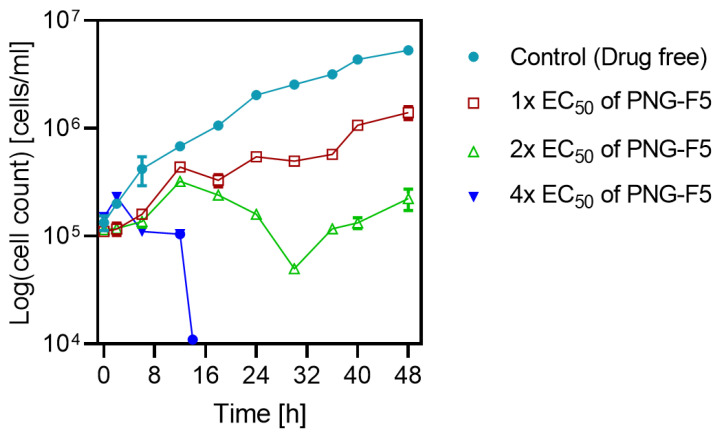
Proliferation of drug-resistant *T. b. brucei* B48 in culture in the presence of 1×, 2× and 4× EC_50_ of hydroxybetulin (EC_50_ = 2.09 µM, Table 4). The final point shown for the 4× EC_50_ concentration was 12 h, after which the cells were not detectable by haemocytometer. The results shown are the average and SD of three determinations. When error bars are not shown they fall inside the symbol.

In order to verify whether or not the most active compound isolated, 20-hydroxybetulin, acted as a trypanocidal or trypanostatic agent, its effect over time on the log phase culture of drug-resistant *T. b. brucei* B48 was tested. A sample of 2 × 10^5^ cells/mL was exposed to concentrations equivalent to 1×, 2× and 4× EC_50_. As a positive control for trypanocidal activity, cells were grown with pentamidine at 1×, 2× and 4× EC_50_; the negative control was a culture grown in the absence of drug. The cell density was determined by counting using a haemocytometer at several time points in triplicate and the average values obtained were plotted against time. The cultures incubated with pentamidine at both 2× and 4× EC_50_ had their growth suppressed and parasites were almost completely eliminated from the media after 32 h. Hydroxybetulin, conversely, had a more trypanostatic effect, as cultures incubated with it at 4 × EC_50_ were still able to grow, although the growth rate was found to be reduced (Figure 1).

## 3. Discussion

In this work, six compounds, two 2-component mixtures and a 3-component mixture were isolated from a propolis sample from the Eastern Highlands of Papua New Guinea. All of the compounds identified were triterpenoids. Profiling of the crude sample by high-resolution LC-MS suggested that it contained many compounds in the triterpenoid class. To our knowledge, there appears to have been no previous investigation of propolis from Papua New Guinea. The abundance of triterpenoids in the sample appears to be typical of samples from other tropical and subtropical regions that we have previously investigated [5,6,25]. The presence of triterpenoids such as mangiferonic and isoferonic acid suggests that one of the sources of the propolis may be mango [5,25]. The crude extract, fractions and isolated compounds and mixtures were tested against *T. b. brucei* WT and a multidrug-resistant strain of *T. b. brucei*, B48. The compounds and mixtures were also tested against *T. congolense* WT and a diminazene-resistant strain [26,27,28]. All the isolated compounds exhibited EC_50_ values of <100 µM against the standard and resistant strains of *T. b. brucei* and *T. congolense*. The most active compound isolated was 20-hydroxybetulin, but the crude extract was more active than any of the isolated compounds apart from 20-hydroxybetulin, suggesting that either the most active compounds were not isolated or there was synergy between the components in the mixture. For all compounds and fractions there was a high degree of selective toxicity against *Trypanosoma* spp. compared to the level of cytotoxicity against a mammalian cell line. The most active compound, hydroxybetulin, had selectivity indices of 23.0 and 48.5 against WT and resistant *T. brucei*, respectively, and 8.1 and 17.0 against WT and resistant *T. congolense,* respectively. Although it was marginally the most toxic compound against mammalian cells, it had by far the highest of the compounds and fractions tested, which justifies its selection as the most promising antiprotozoal compound identified in PNG propolis.

In addition, the crude extract, isolated compounds and fractions were tested against *C. fasciculata* which is a close relative of *Crithidia mellificae* which is a parasite of the honey bee and has been implicated in winter colony collapse [16]. The compounds and fractions were also found to have activity against *C. fasciculata*, although at a lower level than against *Trypanosoma* spp.

Looking at the structures of the isolated compounds in relation to their activity, 20-hydroxybetulin was the most active compound against *T. b. brucei*. It was nearly 19-fold more active than betulin, from which it differs only by the presence of an additional hydroxyl group, and it was 15-fold more active than betulinic acid, where one of the hydroxyl groups in betulin has been converted into a carboxyl group. It was also the most active compound against *T. congolense*—about four times more active than betulin and three-fold more active than betulinic acid. Thus, this suggests that there may be an optimal hydrophilic lipophilic balance for activity, with betulin being insufficiently polar and betulinic acid being too polar for optimal activity. However, this view is slightly confounded by madecassic acid having higher activity against *T. b. brucei* than betulinic acid despite being more polar. There remain many triterpenoids to be isolated from the PNG propolis sample and there may be more active compounds present in the mixture given that its activity was higher than most of the isolated compounds. Importantly, 20-hydroxybetulin was significantly (>2-fold) active against drug-resistant strains of *T. b. brucei* and *T. congolense*, despite the drug resistance mechanisms being very different in the two species [29,30]. Overall, it would seem that 20-hydroxybetulin, which displayed low toxicity against mammalian cell lines, could be a promising lead compound to systematically explore the SAR against African drug-resistant trypanosomiasis.

It is apparent that bees collect propolis to ward off microbial infections and activity has been found against a variety of bee diseases including *Varroa* mite [31], *Paenibacillus larvae* [32] and *Nosema ceranae* [33]. Although bees are not known to ingest propolis, many of the flavonoids present in temperate propolis are found in honey and may originate from propolis gathered by the bees [34]. The presence of a propolis envelope in the bee hive has been found to stabilise and improve the honeybee microbiome [35]. There have been no direct studies on the efficacy of propolis in reducing the burden of protozoa in bee hives. It is well established that the trypanosomatid *Lotmarium passim* is widespread in bee hives [36,37,38,39]. Propolis is frequently antiprotozoal [10] but the extent to which protozoal infection is a threat to bee health has not been established, although the major pathogen *N. ceranae* was once classified as a protozoan.

Recently, compounds isolated from Brazilian propolis have demonstrated activity in vitro against *Leishmania* greater than that shown by the accepted treatment [8]. The bioavailability of propolis in humans is not well established, although there have been some limited studies of temperate propolis where the flavonoids, which are abundant in this type of propolis, appear to be well absorbed but are extensively metabolised [40,41]. However, it might not be necessary for propolis to be absorbed in order to treat protozoal infections such as giardiasis and amoebiasis which infect the gastrointestinal tract [42].

The wide variety of propolis types makes it an attractive source of phytochemicals, particularly since the bee has done much of the work of collecting them in a sustainable way, concentrating them into a solid mass. A recent review identified 578 compounds as having been isolated from honey bee and stingless bee propolis, representing a wide variety of chemical types [43,44]. There are many more compounds, even in the widely studied temperate propolis, left to be fully characterised, and this is even more the case in the variety of tropical propolis samples. Propolis can be collected in large quantities if traps are used in bee hives, and stingless bees can collect very large amounts of propolis [44]. Thus, propolis presents a sustainable source of natural products for drug discovery. The presence of consistent antiprotozoal activity in propolis indicates that it is a promising source of new antiprotozoal drugs. It is still not clear to what extent natural selection plays a part in bees targeting plants with antiprotozoal compounds for propolis collection.

The PNG propolis is typical of some tropical propolis samples, generally from drier and higher regions of the tropics [6]. We tested many of these types of propolis in an earlier paper and found them to be devoid of antioxidant activity [45].

## 4. Materials and Methods

### 4.1. General

The propolis sample was obtained from a collection of samples obtained by Bee Vital, Whitby (Yorkshire, UK). Solvents, reagents and other consumables were obtained from Sigma Aldrich, Fisher Scientific, BioWhittaker or Merck.

### 4.2. Extraction

Approximately 30 g of the propolis sample was extracted thrice under sonication (Clifton ultrasonic bath, Fisher Scientific, Loughborough, UK), with 150 mL of ethanol at room temperature for 60 min. The extracts were combined, and the solvent was evaporated using a rotary evaporator (Buchi, VWR, Leicestershire, UK), and the residue was weighed.

### 4.3. Column Chromatography

About 3 g of the extract was dissolved in 5 mL of ethyl acetate and mixed with 6 g of silica gel in a beaker and allowed to dry in a fume hood. A glass column was packed with 60 g of silica gel 60 (0.063–0.2 mm, Sigma Aldrich) in hexane. The dry adsorbed sample extract was placed directly onto the column and eluted using 200 mL of hexane, ethyl acetate and methanol mixtures as follows: hexane:ethyl acetate (80:20), hexane/ethyl acetate (60:40), hexane/ethyl acetate (40:60), hexane:ethyl acetate (20:80), ethyl acetate and then ethyl acetate/methanol (80:20), ethyl acetate/methanol (60:40), ethyl acetate:methanol (40:60), ethyl acetate:methanol (20:80) and finally methanol to yield fractions F1–F10, respectively.

### 4.4. Purification

Further purification of the column fractions was carried out using MPLC on silica gel using a Grace Reveleris flash chromatography system (Alltech Ltd., Stamford, UK) equipped with evaporative light-scattering detector (ELSD) and UV detector. Fractions F1 and F6 were separately adsorbed onto Celite (1.5 g Sigma Aldrich, UK) and packed into dry loading cartridges. The Reveleris MPLC was set up with a 12 g silica Reveleris column (VWR, Poole, Dorset UK). Fraction 1 (F1) was eluted using the following gradient: 100% hexane 10 min, hexane: ethyl acetate (90:10) 30 min, hexane: ethyl acetate (80:20) 20 min, hexane: ethyl acetate (70:30) 20 min and 100% ethyl acetate for 10 min at a flow rate of 17 mL/min for F1. Fraction F6 was eluted using the following gradient: 100% hexane 10 min, hexane: ethyl acetate (40:60) 30 min, hexane: ethyl acetate (80:20) 20 min, 100% ethyl acetate for 10 min, ethyl acetate: methanol (90:10) 20 min and ethyl acetate: methanol (30:70) for 30 min. Fractions were collected automatically when triggered by the ELSD response. The fractions associated with the same peak according to the ELSD chromatogram were combined, and the solvent was removed and weighed. Fraction F4 was further purified using column chromatography as described above, while F5 was pure and did not require further purification. The purity of the isolated compounds was confirmed by reversed-phase HPLC with ELSD and then characterised by GC–MS, LC–MS and NMR.

### 4.5. HPLC–ELSD and LC-MS Analysis

All samples and fractions were dissolved in methanol to give a concentration of 1 mg/mL and were analysed using an Agilent 1100 HPLC linked to a Shodex ELSD. An ACE C-18 column (150 × 3 mm, 3 μm) with a mobile phase of water (A) and acetonitrile (B) and a flow rate of 0.3 mL/min was used with the following gradient: 25% B for 30 min, 5 min 100% B and 5 min 25% B, injecting 10 μL of sample solution. The high-resolution mass spectra were obtained by running the samples in duplicate using a Dionex 3000 HPLC connected to an Orbitrap Exactive mass spectrometer (ThermoFisher, Hemel Hempstead, UK); the MS detection range was from 100 to 1200 *m*/*z* and the scanning was performed under electrospray ionisation polarity switching mode. The needle voltages were set at −4.0 kV (negative) and 4.5 kV (positive) and sheath and auxiliary gases were at 50 and 17 arbitrary units, respectively. Separation was performed on an ACE C18 column (150 × 3 mm, 3 μm) with 0.1% *v*/*v* formic acid in water as mobile phase A and 0.1% *v*/*v* formic acid in acetonitrile as B at flow rate of 0.300 mL/min using the gradient described for HPLC–ELSD.

### 4.6. GC-MS Analysis

A portion of the extracts and fractions (2 mg) was dissolved in 1 mL of ethyl acetate and 1 μL of each prepared sample was injected in splitless mode at 280 °C into the GC–MS (Focus GC-DSQ2, Thermo Fisher Scientific, Hemel Hempstead, UK) system equipped with a 30 m × 0.25 mm i.d., with 0.25 μm film thickness InertCap 1 MS capillary column from HiChrom (Reading, UK). The temperature gradient was programmed as follows: 100 °C for 2 min, linearly increasing to 280 °C at the rate of 5 °C/min, holding at 280 °C for 15 min and linearly increasing to 320 °C at the rate of 10 °C/min and holding for 10 min. The source temperature was 250 °C and the ionisation voltage was 70 eV for EI–MS.

### 4.7. Nuclear Magnetic Resonance Spectroscopy

About 5–10 mg of the fractions obtained from MPLC purification was dissolved in CDCl_3_ and spectra were acquired using a Bruker AVIII-HD-500 NMR.

### 4.8. Determination of Cytotoxic Effect of PNG Extract and Its Purified Compounds on U937 Mammalian Cells

U937 cells (European Collection of Cell Cultures Cat. No. 85011440, supplied by Sigma Aldrich, Dorset, UK) were cultured as described previously [46]. U937 cells were grown to log phase at 37 °C and harvested at a density of 1 × 10^5^ cells/mL in a 96-well plate (TPP, Trasadingen, Switzerland). Aliquots of 100 µL/well of the cells were added and the plate was incubated for 24 h at 37 °C, 5% CO_2_, 100% humidity. A 2-fold serial dilution of the test compound was carried out in growth medium, in another 96-well plate, and 100 μL of each dilution was then transferred to the cultured cells using a multichannel pipette, followed by another incubation for 24 h. Resazurin dye was then added at a final concentration of 10% (*v*/*v*) and the plates were incubated for a further 24 h, after which fluorescence was measured using a Wallac Victor 2 microplate reader (λ Ex/Em: 560/590 nm). The compounds and fractions were tested in triplicate, and cell viability was expressed as a percentage of the drug-free control. The resulting data were analysed using GraphPad Prism 8 to obtain dose-response curves and corresponding mean inhibitory concentration (EC_50_) values.

### 4.9. Antiprotozoal Assay

The extract and the purified compounds were cultured and tested against *T. b. brucei*, *T. congolense and C. fasciculata* as described previously [6,7]. The *T. b. brucei* strains were a standard drug-sensitive lab strain, Lister 427 (wild-type) [47] and the derived cell line B48 was developed from the wild-type by gene deletion of the drug transporter TbAT1 followed by in vitro adaptation to pentamidine [28], leading to the further loss of the gene encoding TbAQP2 [48], rendering it highly resistant to the diamidine and melaminophenyl arsenical classes of trypanocides. The *T. congolense* strains were the lab strain IL3000 and its diminazene-adapted clone 6C3 [29]. The *C. fasciculata* strain HS6 was a gift of Prof. Terry Smith (University of St Andrews, UK).

### 4.10. Drug Sensitivity Using Cell Count

Different concentrations of hydroxybetulin were tested on the drug-resistant B48 trypanosomes by monitoring in vitro cell growth by using cell count following exposure for different lengths of time. Trypanosomes were taken from cultures at the late logarithmic phase of growth and cell density was determined using a haemocytometer. Cell density was adjusted to the desired concentration of 2 × 10^5^ cells/mL with fresh complete HMI-9 medium. The cell count was taken in triplicate at several time points (0, 4, 8, 12, 16, 24, 32 and 48 h) for different concentrations of the compound and pentamidine, as well as drug-free cells, to serve as a positive control. The experiment was repeated twice and the counts of the three independent determinations were averaged and used for plotting the growth curve.

## Figures and Tables

**Table 1:** Profiling a crude PNG propolis extract using the negative ion masses in LC-MS (RDB = degree of unsaturation).

RT Min.	[M − H]^−^	Formula	RDB	Delta ppm	Intensity
9.4	431.3374	C_24_H_48_O_6_	1.5	0.887	44.3 × 10^6^
10.1	405.2645	C_24_H_38_O_5_	6.5	−0.216	6.58 × 10^6^
14.6	503.3375	C_30_H_48_O_6_	13	−0.432	4.86 × 10^6^
14.9	487.3425	C_30_H_48_O_5_	13	−0.744	6.31 × 10^6^
15.8	489.3218	C_29_H_48_O_6_	7.5	−0.659	1.03 × 10^6^
21.4	485.3265	C_30_H_46_O_5_	8.5	−1.396	1.12 × 10^7^
21.8	471.3473	C_30_H_48_O_4_	7.5	−1.280	2.18 × 10^6^
23.0	473.3630 453.3373	C_30_H_50_O_4_ C_30_H_46_O_3_	7.0 8.5	1.182 −0.237	2.02 × 10^6^ 1.10 × 10^6^
25.5	469.3318 453.3374	C_30_H_46_O_4_ C_30_H_47_O_3_	8.5 8.5	−1.029 0.4355	6.8 × 10^5^ 7.3 × 10^5^
26.2	469.3317 455.3527	C_30_H_46_O_4_ C_30_H_48_O_3_	8.5 7.5	−1.285 −0.760	1.60 × 10^6^
26.9	471.3473	C_30_H_48_O_4_	7.5	−1.343	4.55 × 10^6^
27.9	455.3525	C_30_H_48_O_3_	7.5	−1.095	9.49 × 10^5^
28.7	471.3471	C_30_H_48_O_4_	7.5	−1.653	4.83 × 10^6^
29.1	471.3473 453.3371	C_30_H_48_O_4_ C_30_H_46_O_3_	7.5 8.5	−1.280 −0.641	3.41 × 10^6^
30.3	471.3473	C_30_H_48_O_4_	7.5	−1.343	2.99 × 10^6^
32.9	457.3682	C_30_H_50_O_3_	6.5	−1.003	1.52 × 10 ^6^
35.6	457.3682	C_30_H_50_O_3_	6.5	−0.937	8.5 × 10^5^
36.7	457.3684	C_30_H_50_O_3_	6.5	−0.544	6.63 × 10^6^
38.3	457.3683	C_30_H_50_O_3_	6.5	−0.740	5.33 × 10^6^

**Table 2:** Weights of the fractions obtained from OCC.

Fraction Code	Mass of Fraction (mg)
PNG-F1	934
PNG-F2	301.4
PNG-F3	227.2
PNG-F4	141.8
PNG-F5	54
PNG-F6	307.3
PNG-F7	172.6
PNG-F8	98.3
PNG-F9	52.2
PNG-F10	32.8

**Table 3:** EC_50_ values of PNG propolis and its fractions on *T. b. brucei*. S427 wild-type and B48 (pentamidine-resistant) (*n* = 3).

Samples	*T. b. brucei* S427WT			*T. b. brucei* B48				
	EC_50_ (µg/mL)			EC_50_ (µg/mL)				
	AVG	SD	RSD	AVG	SD	RSD	RF	*t*-Test
PNG crude	4.0	0.095	2.40	3.85	0.37	9.52	0.96	0.50
PNG-F1	10.2	2.50	24.4	5.90	1.28	21.7	0.58	0.06
PNG-F2	14.9	1.27	8.52	9.82	2.61	26.6	0.66	0.040
PNG-F3	8.2	0.73	8.93	5.82	1.38	23.6	0.71	0.05
PNG-F4	4.1	0.27	6.66	2.15	0.51	23.7	0.53	0.005
PNG-F5	2.04	0.11	5.49	2.09	0.11	11.6	0.47	0.003
PNG-F6	4.8	0.60	12.4	2.13	0.50	23.5	0.44	0.004
PNG-F7	15.5	0.98	6.36	7.84	0.70	9.02	0.51	0.0004
PNG-F8	15.8	1.40	8.87	9.16	1.76	19.2	0.58	0.007
PNG-F9	7.9	1.01	12.9	6.55	1.50	22.9	0.83	0.28
PNG-F10	15.8	1.72	9.21	12.1	2.63	21.8	0.76	0.090
Pentamidine ^1^	0.0034	0.0008	22.2	0.721	0.050	6.75	210	0.00001

Abbreviations: AVG EC_50_ = average of half maximal effective concentration, average of at least 3 independent determinations. SD = standard deviation of all determinations. RSD = relative standard deviation = (SD/Avg.) × 100. RF = resistance factor ((EC_50_ B48/EC_50_) WT). Statistical significance was determined using an unpaired two-tailed Student’s *t*-test comparing EC_50_ value of the resistant strain with that of the same sample for the control strain S427. ^1^ values of EC_50_ and SD in µM.

**Table 4:** IC_50_ values of crude PNG propolis extract and its fractions against U937 cells (*n* = 3).

Samples	AVG EC_50_ (μg/mL)		
		SD	RSD (%)
PNG crude	116.3	5.7	4.90
PNG-F1	83.3	11.1	13.3
PNG-F2	135.1	20.1	14.9
PNG-F3	84.9	5.0	5.8
PNG-F4	40.1	1.5	3.7
PNG-F5	47.0	9.63	20.5
PNG-F6	84.4	9.5	11.3
PNG-F7	57.0	6.8	11.8
PNG-F8	95.6	2.3	2.4
PNG-F9	45.9	2.0	4.4
PNG-F10	53.4	5.2	9.8

SD = standard deviation; RSD (%) = relative standard deviation = (SD/Avg.) × 100.

**Table 5:** EC_50_ values of purified compounds isolated from PNG propolis on *T. b. brucei* s427 WT and *T. b. brucei* B48 (*n* = 3).

Samples	*T. b. brucei* S427WT				*T. b. brucei* B48					
	AVG EC_50_				AVG EC_50_					
	μg/mL	μM	SD	RSD	μg/mL	μM	SD	RSD	RF	*t*-Test
Hydroxybetulin	2.04	4.44	0.11	5.49	0.97	2.09	0.11	11.6	0.47	0.0003
PNG-F6-S12	16.0	-	1.47	9.21	11.3	-	2.54	22.5	0.71	0.051
PNG-F6-S15	13.5	-	0.84	6.22	12.4	-	0.62	5.02	0.91	0.12
Hydroxymangi-ferolic acid	13.6	28.8	1.45	10.7	11.8	25.0	2.46	20.8	0.87	0.33
PNG-F1-5B	18.5	-	0.72	3.90	17.6	-	0.68	3.84	0.95	0.21
Betulin	26.6	60.1	2.69	6.26	22.3	50.5	2.39	11.8	0.84	0.13
Betulinic acid	24.2	53.1	2.64	12.7	19.6	42.9	1.66	8.50	0.81	0.0610
Madecassic acid	18.1	36.0	1.81	9.45	16.0	31.7	0.91	10.4	0.88	0.23
Pentamidine	-	0.0043	0.0020	47.3	-	0.62	0.10	16.5	145	0.0005

**Table 6:** EC_50_ values of PNG propolis and its fractions on *T. congolense* IL3000, and *T. congolense* resistant to diminazene (*n* = 3).

Samples	*T. congolense* IL3000			*T. congolense* 6C3				
	AVG EC_50_			AVG EC_50_				
	μg/mL	SD	RSD	μg/mL	SD	RSD	RF	*t*-Test
PNG crude	3.39	0.41	12.3	4.4	0.37	8.52	1.29	0.039
PNG-F1	13.5	3.26	24.3	13.3	3.40	25.6	0.99	0.95
PNG-F2	11.4	2.49	21.8	12.0	2.34	19.5	1.05	0.79
PNG-F3	11.5	2.93	25.6	12.8	2.65	20.7	1.11	0.59
PNG-F4	10.5	2.41	23.0	9.3	0.79	8.6	0.89	0.46
PNG-F5	5.77	1.28	22.2	6.09	0.33	11.8	0.48	0.017
PNG-F6	9.30	1.64	17.7	7.8	0.69	8.88	0.84	0.017
PNG-F7	12.6	1.42	11.2	12.4	2.55	20.5	0.98	0.22
PNG-F8	15.6	3.10	19.9	14.6	2.64	18.1	0.93	0.90
PNG-F9	18.1	2.14	11.8	20.5	3.05	14.9	1.13	0.69
PNG-F10	18.2	3.94	21.7	21.2	4.17	19.6	1.17	0.34
Diminazene ^1^	0.26	0.028	11.0	1.54	0.22	14.2	5.90	0.0005

^1^ EC_50_ and SD given in µM.

**Table 7:** EC_50_ values of purified compounds isolated from PNG propolis against drug-sensitive *T. congolense* IL3000, and diminazene-resistant *T. congolense* 6C3 (*n* = 3).

Samples	*T. congolense* IL3000				*T. congolense* 6C3					
	AVG EC_50_				AVG EC_50_					
	μg/mL	μM	SD	RSD	μg/mL	μM	SD	RSD	RF	*t*-Test
Hydroxybetulin	5.77	14.4	1.28	22.2	2.77	6.09	0.33	11.8	0.48	0.017
PNG-F6-S12	14.2	-	2.75	19.4	16.9	-	4.00	23.5	1.18	0.40
PNG-F6-S15	13.7	-	2.75	20.1	13.2	-	2.83	21.4	0.96	0.83
Hydroxymangi-ferolic acid	16.7	35.3	3.49	20.9	15.7	33.2	3.62	23.1	0.94	0.75
PNG-F1-5	18.9		2.90	15.3	19.5		4.00	20.5	1.03	0.85
Betulin	21.5	70.1	3.15	14.7	22.1	49.9	3.46	15.7	1.03	0.84
Betulinic acid	17.2	44.5	2.93	17.0	18.1	28.5	3.73	20.6	1.05	0.75
Madecassic acid	22.6	67.6	3.25	14.4	19.8	59.1	5.37	27.1	0.88	0.48
Diminazene	-	0.28	0.019	6.9	-	1.55	0.22	14.2	5.5	0.0006

**Table 8:** IC_50_ of cytotoxicity of isolated purified compounds from PNG propolis against U937 cells (*n* = 3).

Samples	U937 Cells				
	AVG EC_50_				
	μg/mL	μM	SD	RSD	SI ^1^
Hydroxybetulin	47.0	102.0	9.63	20.50	23.0
PNG-F6-S12	>100	N/A	9.55	8.92	>6.3
PNG-F6-S15	>100	N/A	12.30	8.44	>7.4
Hydroxymangiferolic acid	>100	260.1	7.42	6.04	>7.4
PNGF1-5	92.7	N/A	6.24	6.73	5.0
Betulin	55.8	126.0	4.34	7.78	2.1
Betulinic acid	51.7	113.1	4.85	9.38	2.1
Madecassic acid	90.7	179.8	5.50	6.06	5.0

^1^ SI, selectivity index relative to *T. b. brucei* WT: EC_50_(U937)/EC_50_(TbbWT).

**Table 9:** EC_50_ of PNG propolis and its fractions on *C. fasciculata*.

Exp Code	AVG EC_50_ (μg/mL)	SD	RSD
PNG crude	20.8	1.3	6.0
PNG-F1	22.1	4.44	20.1
PNG-F2	34.3	2.80	8.15
PNG-F3	28.1	1.96	7.00
PNG-F4	20.8	2.73	13.1
PNG-F5	23.4	3.15	13.5
PNG-F6	53.1	4.35	8.20
PNG-F7	38.5	5.67	14.7
PNG-F8	31.3	5.87	18.8
PNG-F9	24.7	1.17	4.74
PNG-F10	32.2	2.29	7.10
PAO (µM)	3.06	0.08	2.47

## Data Availability

All relevant data are contained in the manuscript and Supplemental Information.

## Supplementary Materials

The following supporting information can be downloaded online. Figure S1: Work Flow, Figure S2 Cycloartenol, Figure S3 Methylenecycloartenol, Figure S4 Cycloeucalenol, Figure S5 GC-MS analysis of cycloartenol (13.16 min.), 24 (28)-methylenecycloartenol (13.43 min.) and cycloeucalenol (13.61 min.) in fraction PNG-F1-5B. Table S1 ^1^H (500 MHz), ^13^C (100 MHz) data of compounds cycloartenol, methylene cycloartenol and cycloeucalenol in fraction PNG-F1-5B. Figure S6 ^1^H NMR (500 MHz) spectrum of the mixture of cycloartenol, 24 (28)-methylenecycloartenol and cycloeucalenol in CDCl_3_. Figure S7 Dept q (100 MHz) spectrum of the mixture of cycloartenol, 24 (28)-methylenecycloartenol and cycloeucalenol in CDCl_3_. Figure S8 HSQC (500 MHz) spectrum of the mixture of cycloartenol, 24 (28)-methylenecycloartenol and cycloeucalenol in CDCl_3_. Figure S9 COSY (500 MHz) Spectrum of the mixture of cycloartenol, 24 (28)-methylenecycloartenol and cycloeucalenol in CDCl_3_. Figure S10 Structure of betulin. Table S2 ^1^H (500 MHz), ^13^C (100 MHz) data of betulin. Figure S11 ^1^H NMR (500 MHz) spectrum of betulin in CDCl_3_. Figure S12 ^13^C NMR (100 MHz) spectrum of betulin in CDCl_3_. Figure S13 Structure of betulinic acid. Table S3 ^1^H (500 MHz), ^13^C (100 MHz) data of betulinic acid. Figure S14 ^1^H NMR (500 MHz) spectrum of betulinic acid in DMSO-d6. Figure S15 Dept q (100 MHz) spectrum of betulinic acid in DMSO-d6. Figure S16 Structure of madecassic acid Table S4 ^1^H (500 MHz), ^13^C (100 MHz) data of madecassic acid. Figure S17 ^1^H NMR (500 MHz) spectrum of madecassic acid in DMSO-d6. Figure S18 Dept q (100 MHz) spectrum of madecassic acid in DMSO-d6. Figure S19 Structure of 20-hydroxybetulin. Table S5: ^1^H (500MHz), ^13^C (100MHz) data 20-hydroxybetulin. Figure S20 ^1^H NMR (500 MHz) spectra of 20-hydroxybetulin in CDCl_3._ Figure S21 Dept q (100 MHz) spectrum of 20-hydroxybetulin in CDCl_3_. Figure S22 Structure of mangiferonic acid. Figure S23 Structure of ambonic acid. Table S6 ^1^H (500 MHz), ^13^C (100 MHz) data for mangiferonic acid and ambonic acid. Figure S24 ^1^H NMR (500 MHz) spectrum of mangiferonic acid and ambonic acid in CDCl_3._ Figure S25 Dept q (100 MHz) spectrum of the mixture of mangiferonic acid and ambonic acid in CDCl_3._ Figure S26 Structure of isomangiferolic acid. Figure S27 Structure of ambolic acid. Table S7 ^1^H (500 MHz), ^13^C (100 MHz) data for isomangiferolic acid and ambolic acid. Figure S28 ^1^H NMR (500 MHz) spectrum of the mixture of isomangiferolic acid and ambolic acid in CDCl_3_. Figure S29 Dept q (100 MHz) spectrum of the mixture of isomangiferolic acid and ambolic acid in CDCl_3_. Figure S30 Structure of 27-Hydroxyisomangiferolic acid. Table S8 ^1^H (500 MHz), ^13^C (100 MHz) data for 27-Hydroxyisomangiferolic acid. Figure S31 ^1^H NMR (500 MHz) spectrum for 27-Hydroxyisomangiferolic acid in DMSO-d6. Figure S32 Dept q (100 MHz) spectrum of 27-Hydroxyisomangiferolic acid in DMSO-d6.

## Sample Availability

Samples of the compounds can be obtained from the authors subject to availability.
