# Supplementary material for: The Antiprotozoal Activity of Papua New Guinea Propolis and Its Triterpenes
Source: Molecules. 2022 Mar 1;27(5):1622. doi: 10.3390/molecules27051622 (PMC8911803; doi:10.3390/molecules27051622)
Supplement: Supplementary file 1 [file molecules-27-01622-s001.zip › molecules-1603370-SI.pdf]

Article

The Antiprotozoal Activity of Papua New Guinea Propolis and Its Triterpenes

Samya S. Alenezi [1], Naif D. Alenezi [1], Godwin U. Ebiloma [2,3], Manal J. Natto [2], Marzuq A. Ungogo [2], John O. Igoli [2,4], Valerie A. Ferro [1], Alexander I. Gray [1], James Fearnley [5], Harry P. de Koning [2,*] and David G. Watson [1,*]

1 Strathclyde Institute of Pharmacy and Biomedical Science, University of Strathclyde, 161 Cathedral Street, Glasgow G4 0RE, UK; sameahq8@gmail.com (S.S.A.); ndenzi@sfda.gov.sa (N.D.A.); v.a.ferro@strath.ac.uk (V.A.F.); a.i.gray345@gmail.com (A.I.G.)

2 Institute of Infection, Immunity and Inflammation, College of Medical, Veterinary and Life Sciences, University of Glasgow, Glasgow G12 8TA, UK; g.ebiloma@tees.ac.uk (G.U.E.); manal.natto@glasgow.ac.uk (M.J.N.); 2226184u@student.gla.ac.uk (M.A.U.); j.o.igoli@uam.edu.ng (J.O.I.)

3 School of Health and Life Sciences, Teesside University, Middlesbrough TS1 3BX, UK

4 Phytochemistry Research Group, Department of Chemistry, University of Agriculture, Makurdi PMB 2373, Nigeria

5 BeeVital, Whitby, North Yorkshire YO22 5JR, UK; james.fearnley@beevitalpropolis.com

* Correspondence: harry.de-koning@glasgow.ac.uk (H.P.d.K.); d.g.watson@strath.ac.uk (D.G.W.)

Figure S1 Work Flow

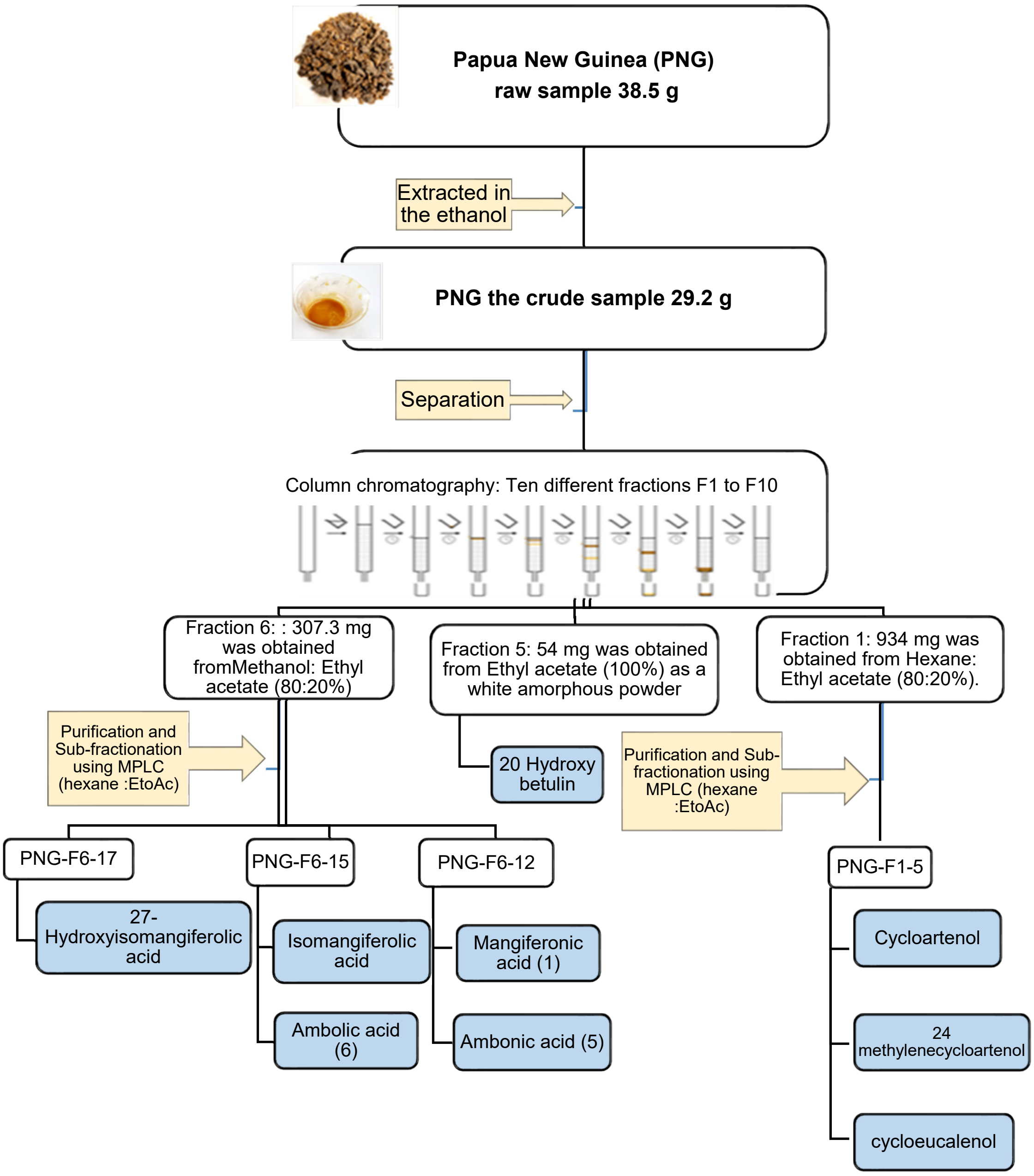

__S1__ Characterization of PNG-F1-5 as mixture of Cycloartenol, 24 (28)-Methylenecycloartenol and Cycloeucalenol

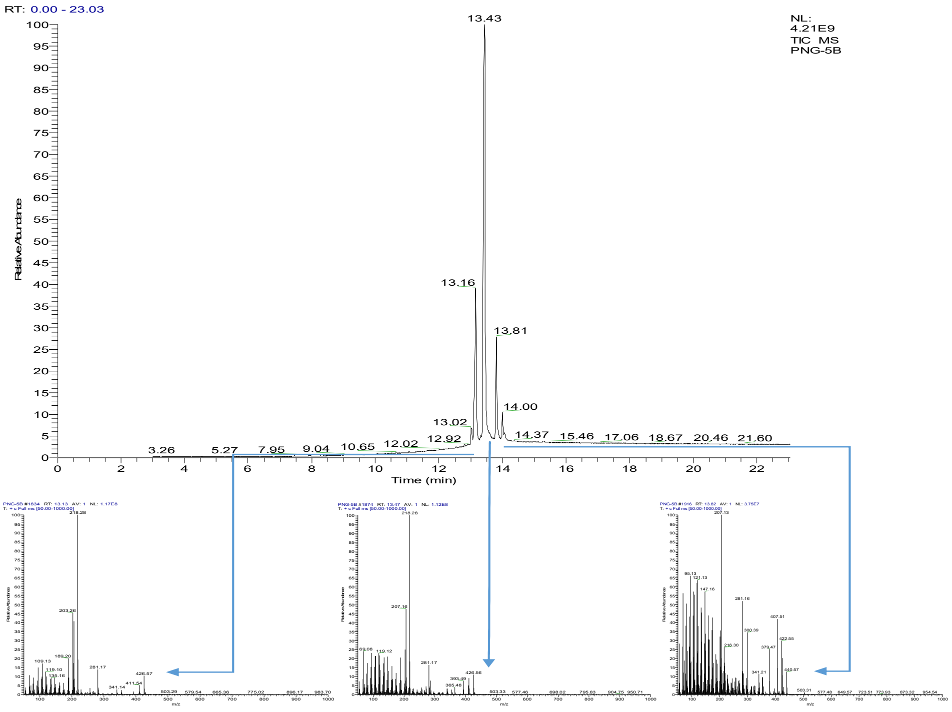

Figure S2 GC-MS analysis of cycloartenol (13.16 min), 24 (28)-methylenecycloartenol (13.43 min.) and cycloeucalenol (13.61 min.). in fraction PNG-F1-5B

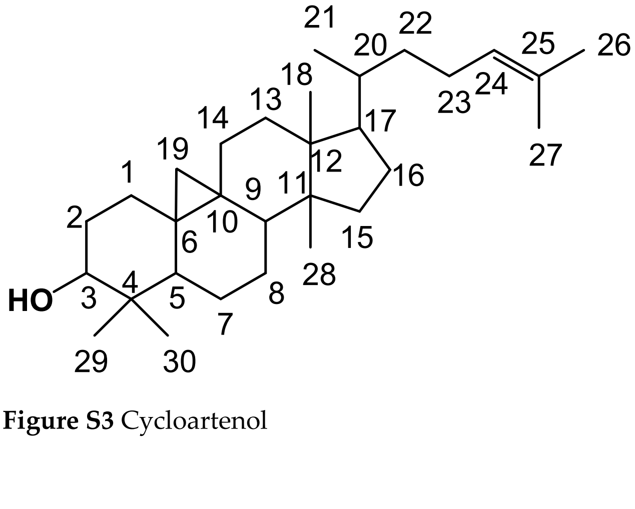

Figure S3 Cycloartenol

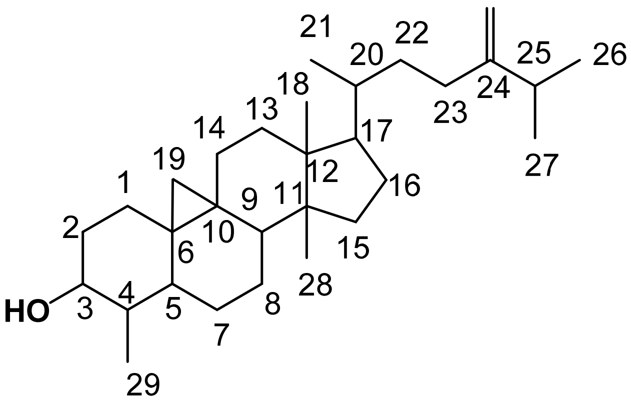

Figure S4 24-Methylenecycloartanol

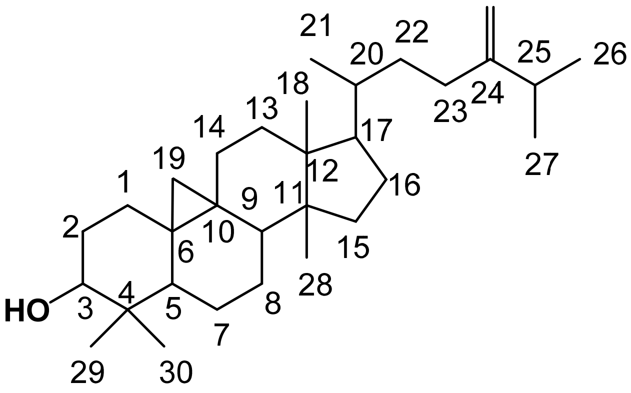

Figure S5 Cycloeucalenol

-

Table S1 ^{1}H (500MHz), ^{13}C (100MHz) data for the mixture of cycloartenol, methylene cycloartenol and cycloeucalenol.

Position	Compound					
	Cycloartenol		24 (28)-methylenecycloartenol		Cycloeucalenol	
	^{1}H δ ppm (mult, *J*)	^{13}C δ ppm	^{1}H δ ppm (mult, *J*)	^{13}C δ ppm	^{1}H δ ppm (mult, *J*)	^{13}C δ ppm
1	1.55 1H (m), 1.28 1H (m)	32.0 (CH_2)	1.55 1H (m), 1.28 1H (m)	32.0 (CH_2)	1.55 1H (m), 1.28 1H (m)	30.4 (CH_2)
2	1.92 1H (m), 1.65 1H (m)	26.5 (CH_2)	1.92 1H (m), 1.65 1H (m)	26.6 (CH_2)	1.83 1H (m) 1.59 1H (m)	27.0 (CH_2)
3	3.31 (m)	78.9 (CH)	3.26 (m)	79.1 (CH)	3.22 (m)	79.0 (CH)
4	-	40.5 (C)	–	40.0 (C)	1.32 (m)	41.5 (C)
5	1.32 1H (m)	39.6 (CH)	1.32 1H (m)	39.7 (CH)	1.32 1H (m)	41.8 (CH)
6	1.65 1H (m), 0.81 1H (m)	21.1 (CH_2)	1.65 1H (m), 0.81 1H (m)	21.1 (CH_2)	1.65 1H (m), 0.81 1H (m)	20.9 (CH_2)
7	1.15 1H (m), 1.32 1H (m)	26.0 (CH_2)	1.15 1H (m), 1.32 1H (m)	26.0 (CH_2)	1.15 1H (m), 1.32 1H (m)	26.5 (CH_2)
8	1.52 1H (m)	47.1 (CH)	1.52 1H (m)	47.1 (CH)	1.52 1H (m)	47.2 (CH)
9	-	20.1 (C)	–	19.9 (C)	-	23.6 (C)
10	-	26.0 (C)	–	26.1 (C)	-	26.2 (C)
11	1.07 1H (m), 0.81 (m)	28.1 (CH_2)	1.07 1H (m), 0.81 (m)	28.1 (CH_2)	1.07 1H (m), 0.81 (m)	28.1 (CH_2)
12	1.59 1H (m), 1.28 1H (m)	32.9 (CH_2)	1.59 1H (m), 1.28 1H (m)	35.7 (CH_2)	1.59 1H (m), 1.28 1H (m)	32.9 (CH_2)
13	-	45.2 (C)	–	45.2 (C)	-	45.3 (C)
14	-	48.7 (C)	–	49 (C)	-	49.0 (C)
15	1.33 1H (m), 0.90 1H (m)	32.9 (CH_2)	1.33 1H (m), 0.90 1H (m)	32.9 (CH_2)	1.33 1H (m), 0.90 1H (m)	32.9 (CH_2)
16	2.02 1H (m), 1.32 1H (m)	71.8 (CH_2)	2.02 1H (m), 1.32 1H (m)	26.5 (CH_2)	2.02 1H (m), 1.32 1H (m)	28.6 (CH_2)
17	1.65 1H (m)	52.3 (CH)	1.65 1H (m)	52.5 (CH)	1.65 1H (m)	52.2 (CH)
18	0.99 3H (s)	18.2 (CH_3)	0.99 1H (s)	18 (CH_3)	0.99 1H (s)	17.8 (CH_3)
19	0.58 1H (d, 4.21), 0.35 1H (d, 4.18)	29.7 (CH_2)	0.58 1H (d, 4.21), 0.35 1H (d, 4.18)	29.9 (CH_2)	0.58 1H (d, 4.21), 0.35 1H (d, 4.18)	29.9 (CH_2)
20	1.40 1H (m)	30.3 (CH)	1.40 1H (m)	36 (CH)	1.40 1H (m)	36.0 (CH)
21	0.92 3H (s)	18.0 (CH_3)	0.92 3H (s)	18.3 (CH_3)	0.92 3H (s)	18.4 (CH_3)
22	1.33 2H (m)	35.5 (CH_2)	1.33 2H (m)	35.6 (CH_2)	1.33 2H (m)	35.6 (CH_2)
23	1.77 1H (m), 0.97 1H (m)	24.9 (CH_2)	2.02 1H (m), 1.65 1H (m)	31.2 (CH_2)	2.23 1H (m), 1.65 1H (m)	31.1 (CH_2)

24	5.21 IH (t, 3.68)	121.7 (CH)	-	156.9 (C)	-	156.5 (C)
25	-	130.7 (C)	2.33 (m)	33.8 (CH)	2.33 (m)	33.9 (CH)
26	1.71 3H (s)	26.1 (CH_3)	1.28 3H	21.8 (CH_3)	1.026 d	22.1 (CH_3)
27	1.63 3H (s)	18.2 (CH_3)	1.02 3H (s)	23.7 (CH_3)	1.03 3H (s)	23.3 (CH_3)
28	0.90 3H (s)	19.3 (CH_3)	0.90 3H (s)	19.1 (CH_3)	0.90 3H (s)	19.1 (CH_3)
29	0.83 3H (s)	14.0 (CH_3)	0.83 3H (s)	10.1 (CH_3)	0.83 3H (s)	14.46 (CH_3)
30	0.99 3H (s)	25.5 (CH_3)	0.99 3H (s)	25.7 (CH_3)	4.59 1H (dd,, 2.74, 1.38) 4.71 1H (d, 2.64)	106.9 (CH_2)
31			4.69 IH (d, 1.59) 4.74 1H (s)	109.3 (CH_2)		

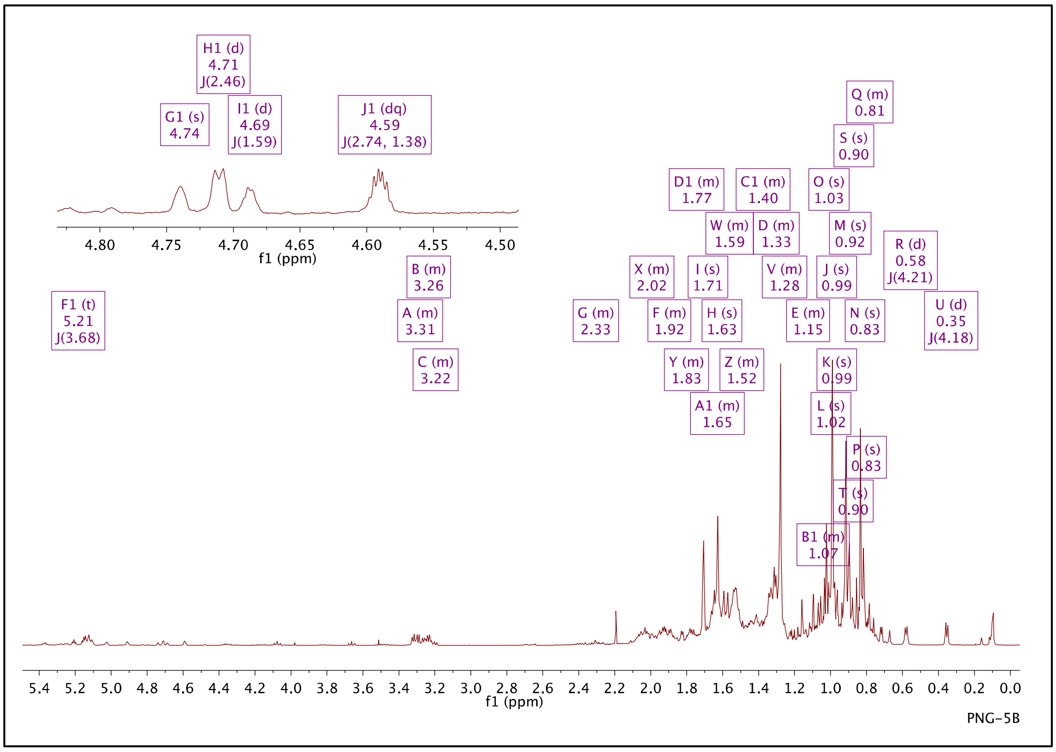

Figure S6 ¹H NMR (500 MHz) spectrum of the mixture of cycloartenol, 24 (28)-cethylenecycloartenol and cycloeucalenol in $CDCl_3$

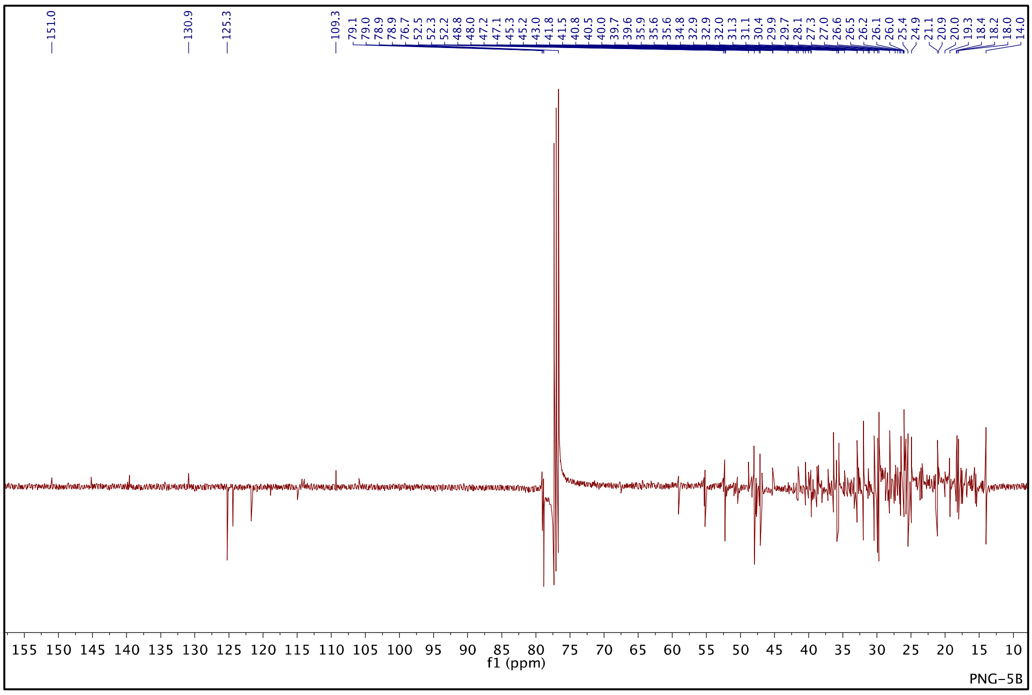

Figure S7 Dept q NMR (100 MHz) spectrum of the mixture of cycloartenol, 24 (28)-methylenecycloartenol and cycloeucalenol in $CDCl_3$

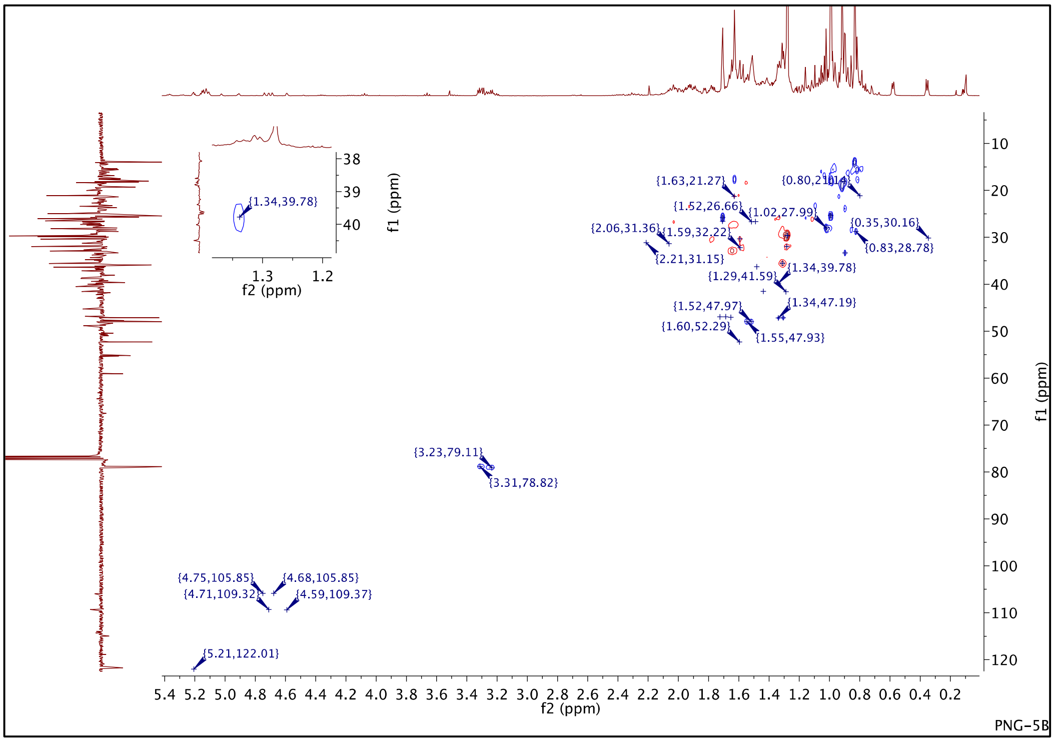

Figure S8 HSQC (500 MHz) spectrum of the mixure of cycloartenol, 24 (28)-methylenecycloartenol and cycloeucalenol in $CDCl_3$

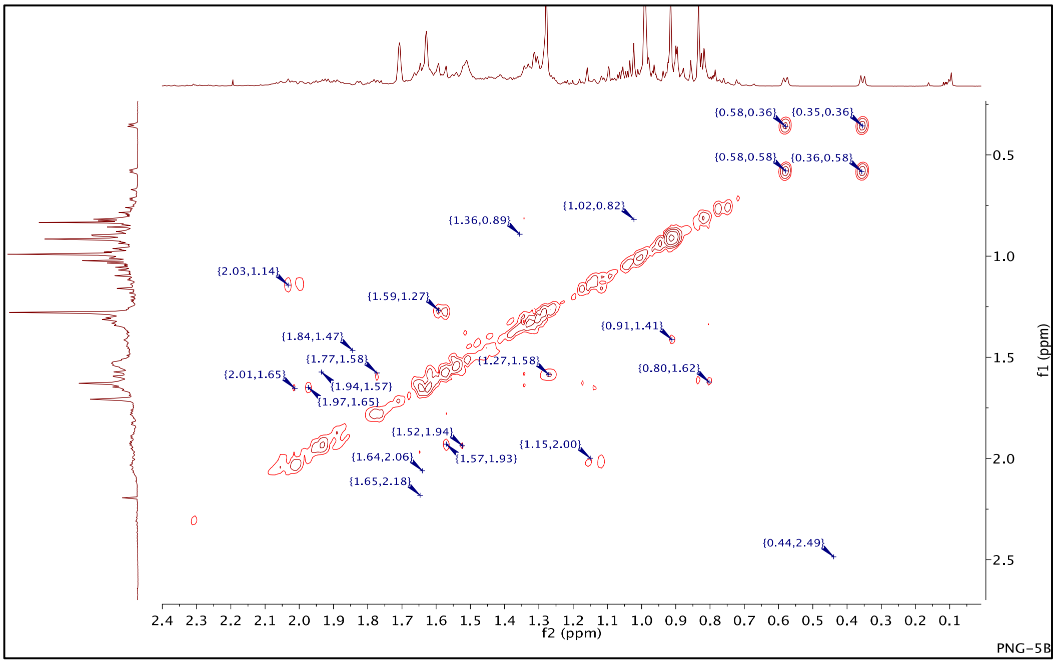

Figure S9 COSY (500 MHz) Spectrum of the mixture of cycloartenol, 24 (28)-methylenecycloartenol and cycloeucalenol in $CDCl_3$

S2 Characterisation of PNG-F4-11 as Betulin

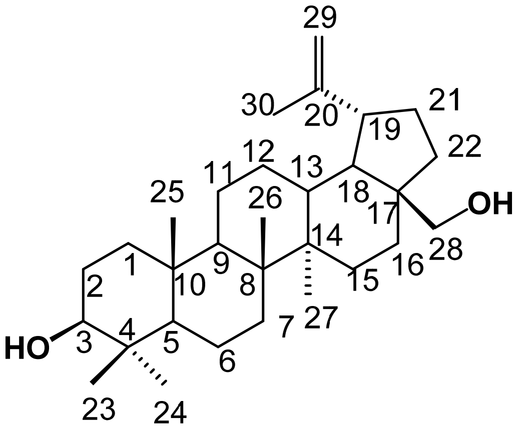

Figure S10 Structure of betulin

Table S2: ^{1}H (500MHz), ^{13}C (100MHz) data of betulin

Position	Compound	
	(10)<br>Betulin	
	^{1}H δ ppm (mult, *J* (Hz))	^{13}C δ ppm (mult)
1	1.66 1H (m) 0.89 1H (m)	37.3 (CH_2)
2	1.57 1H (m) 1.07 1H (m)	28.0 (CH_2)
3	3.19 1H (dd, *J* = 11.4, 4.8)	79.0 (CH)
4	-	38.7 (C)
5	0.68 1H (m)	55.3 (CH)
6	1.52 1H (dt *J* = 11.1, 4.1) 1.41 1H (m)	18.4 (CH_2)
7	1.41 2H (m)	34.2 (CH_2)
8	-	38.9 (C)
9	1.27 1H (m)	50.4 (CH)
10	-	37.2 (C)
11	1.41 2H (m)	20.8 (CH_2)
12	1.66 1H (m) 1.07 1H (m)	25.2 (CH_2)
13	1.66 1H (m), 0.97 1H (m)	28.0 (CH)
14	-	40.9 (C)
15	1.61 1H (m), 1.07 1H (m)	27.1 (CH_2)
16	1.95 1H (m), 1.27 1H (m)	29.2 (CH_2)
17	-	42.7 (C)
18	1.57 1H (m),	48.8 (CH)
19	2.38 1H (td, *J* = 11.0, 5.8)	47.8 (CH)
20	-	150.5 (C)

21	1.411H (m), 1.20 1H (m)	29.8 (CH_2)
22	1.85 1H (ddd, *J* = 12.3, 8.5. 1.3) 1.07 1H (m)	34.0 (CH_2)
23	0.97 3H (s)	28.0 (CH_3)
24	0.76 3H (s)	15.4 (CH_3)
25	0.82 3H (s)	16.1 (CH_3)
26	0.98 3H (s)	16.0 (CH_3)
27	1.02 3H (s)	14.8 (CH_3)
28	3.80 1H (dd, *J* = 10.8, 1.9)<br>3.33 1H (d *J* = 10.6)	60.6 (CH_2)
29	4.68 1H (d *J* = 2.2)<br>4.58 1H (m)	109.7 (CH_2)
30	1.68 3H (s)	19.1 (CH_3)

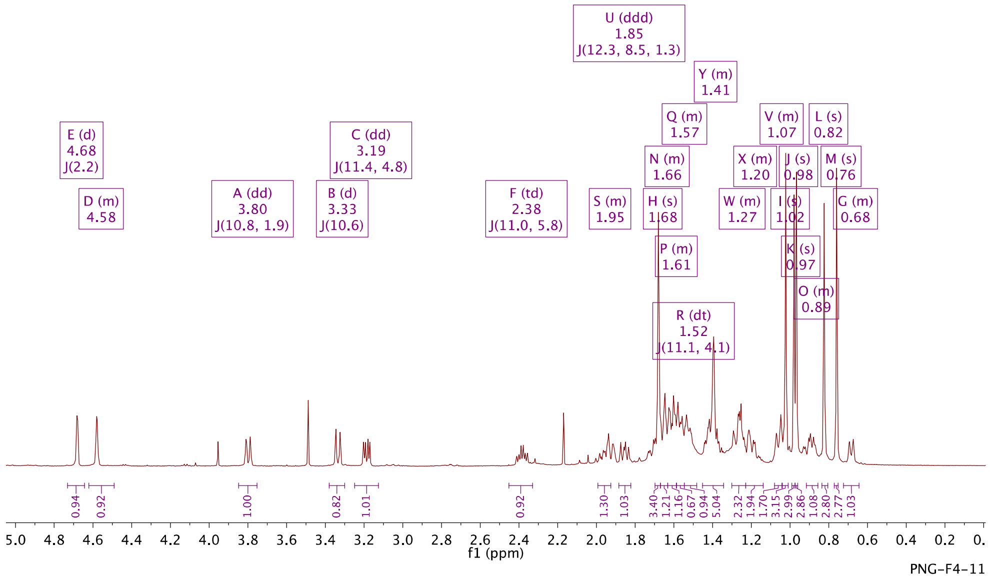

Figure S11 ^{1}H NMR (500 MHz) spectrum of betulin in $CDCl_3$

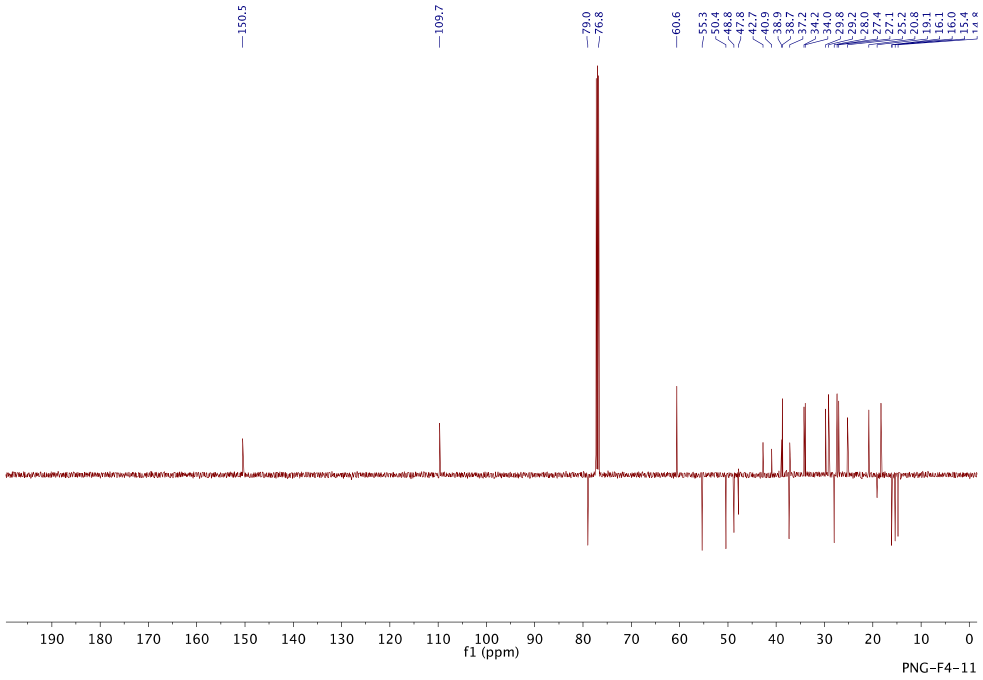

Figure S12 Dept q NMR (100 MHz) spectrum of betulin in $CDCl_3$

S3 Characterisation of PNG-F4-13 as Betulinic acid

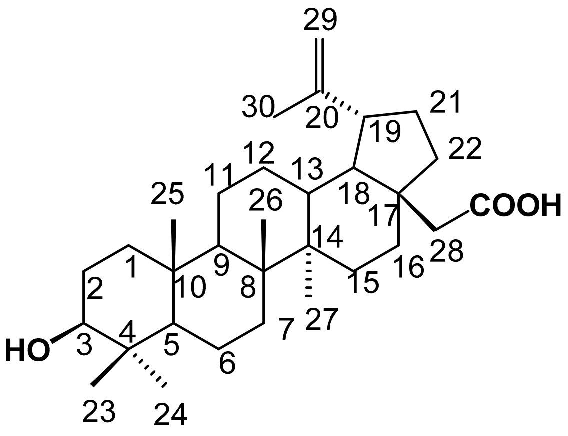

S13 Structure of betulinic acid

Table S3 ^{1}H (500MHz), ^{13}C (100MHz) data of betulinic acid **.**

Position	^{1}H	^{13}C
	δ ppm (mult, *J* (Hz))	δ ppm (mult)
1	1.57 1H (m) 0.81 1H (m)	38.3 (CH_2)
2	1.43 2H (m)	27.2 (CH_2)
3	2.95 1H (ddt, *J* = 16.5, 11.2, 5.6)	76.8 (CH)
4	-	38.5 (C)
5	0.62 1H (m)	54.9 (CH)
6	1.43 1H (m) 1.36 1H (m)	18.0 (CH_2)
7	1.36 2H (m)	33.9 (CH_2)
8	-	40.3 (C)
9	1.25 1H (dd, *J* = 12.7, 2.5)	49.9 (CH)
10	-	36.7 (C)
11	1.36 1H (m) 1.14 1H (m)	20.5 (CH_2)
12	1.57 1H (m) 1.09 1H (m)	25.1 (CH_2)
13	2.21 1H (td, *J* = 12.3, 3.5)	37.6 (CH)
14	-	42.0 (C)
15	1.79 1H (q, *J* = 8.2, 6.8), 1.31 1H (m)	30.1 (CH_2)
16	2.101H (m), 1.36 1H (m)	31.7 (CH2)
17	-	55.4 (C)
18	1.50 1H (d, *J* = 11.3)	46.6 (CH)
19	2.95 1H (ddt, *J* = 16.5, 11.2, 5.6)	48.6 (CH)
20	-	150.4 (C)
21	1.31 1H (m), 1.08 1H (m)	29.2 (CH_2)
22	1.79 1H (q, *J* = 8.2, 6.8) 1.43 1H (m)	36.3 (CH_2)
23	0.86 3H (s)	28.1 (CH_3)
24	0.65 3H (s)	15.7 (CH_3)
25	0.76 3H (s)	16.0 (CH_3)
26	0.86 3H (s)	15.8 (CH_3)
27	0.93 3H (s)	14.5 (CH_3)
28	-	177.2 (C)
29	4.68 1H (d, *J* = 2.5)<br>4.56 1H (s)	109.6 (CH_2)
30	1.64 3H (s)	19.0 (CH_3)

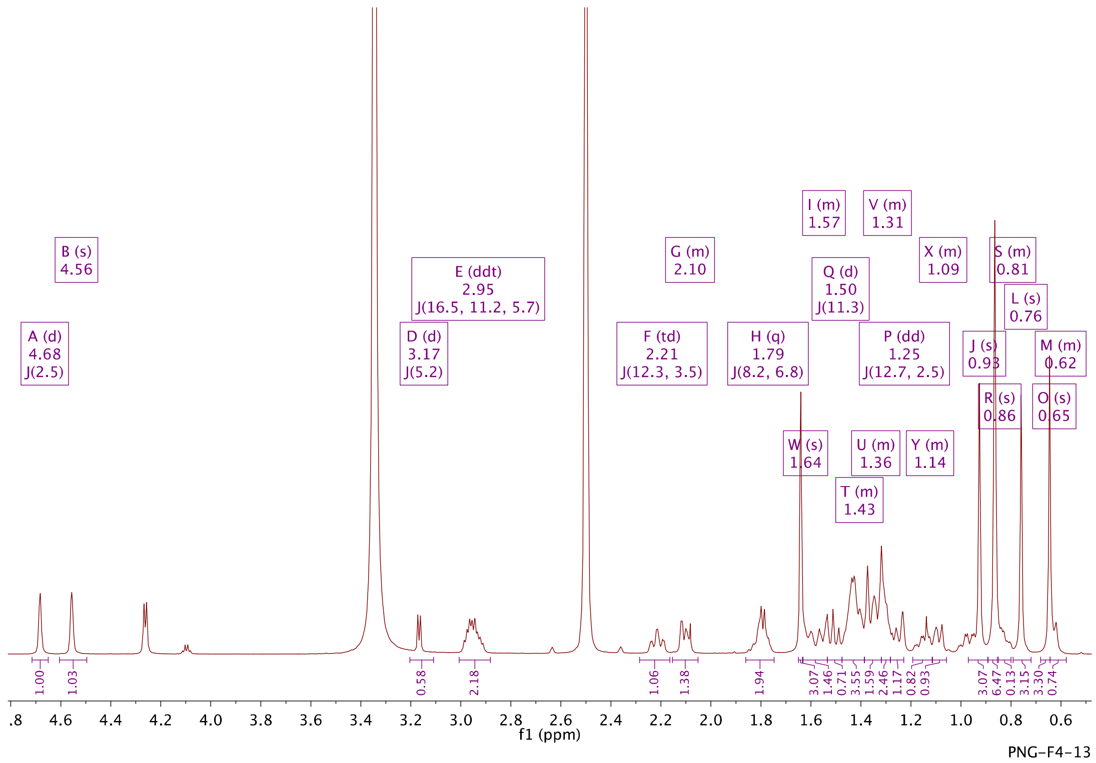

Figure S14 ^{1}H NMR (500 MHz) spectrum of betulinic acid in DMSO-d6.

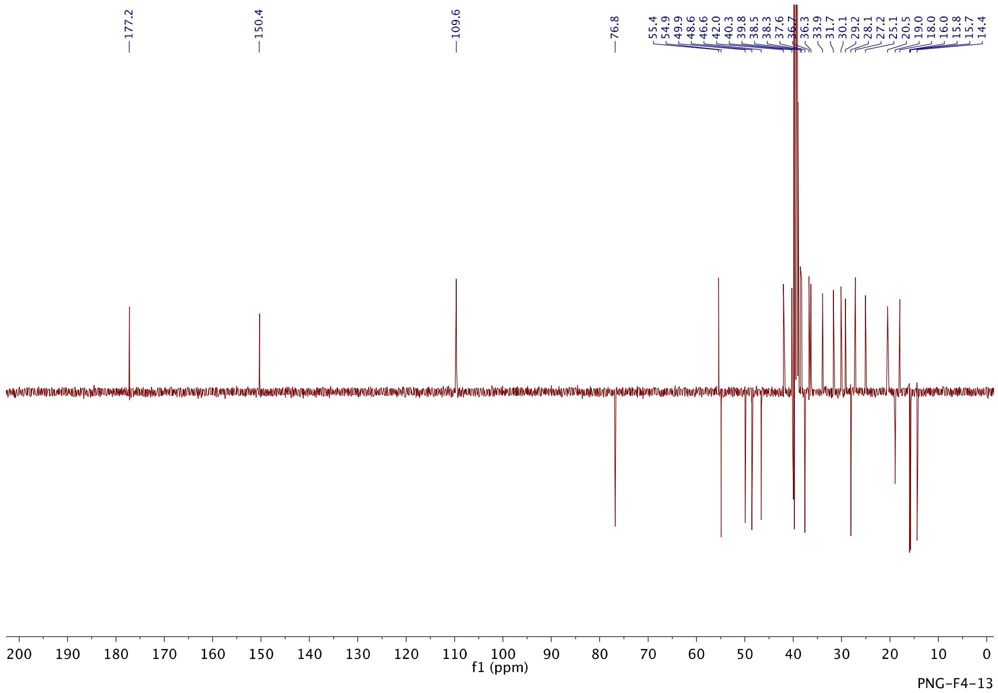

Figure S15 Dept q NMR (100 MHz) spectrum of betulinic acid in DMSO-d6.

S4 Characterization of PNG-F4-18 as Madecassic acid

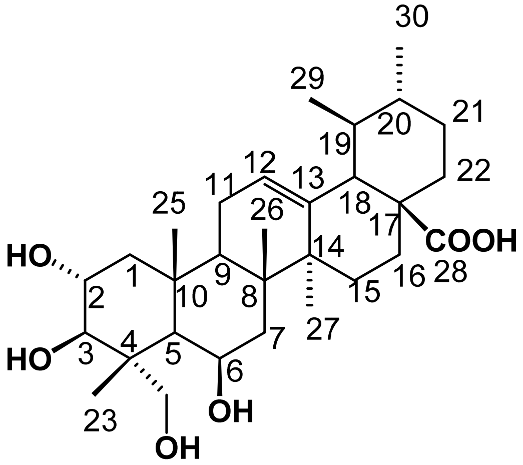

Figure S16 Structure of madecassic acid

Table S4 ^{1}H (500MHz), ^{13}C (100MHz) data of madecassic acid.

Position	Compound	
	**(12)**<br>**Madecassic acid**	
	**^{1}H δ ppm (mult, *J*, (Hz))**	**^{13}C δ ppm (mult)**
1	0.71 1H, (t, *J* = 11.9)<br>1.72 1H (dd, *J* = 12.5, 4.6)	49.2 (t)
2	3.51 (1H, m)	67.5 (d)
3	3.11 (1H, d, *J* = 9.4 Hz)	76.2 (d)
4	-	43.4 (s)
5	1.15 1H (d, *J* = 1.8 Hz)	47.0 (d)
6	4.25 (1H, m)	65.9 (CH)
7	1.58 (1H, m), 1.37 (1H, m)	40.0 (CH_2)
8	-	42.2 (C)
9	1.52 (1H, m)	47.3 (CH)
10	-	38.3 (C)
11	1.95 1H (m), 1.52 1H (m)	23.4 (CH_2)
12	5.17 (q, 4.2)	124.9 (CH)
13	-	137.7 (C)
14	-	42.6 (C)
15	1.87 1H, (m)<br>0.95 1H, (t, *J* = 11.9)	28.1 (CH_2)
16	1.95 1H (m), 1.02 1H (m)	23.8 (CH_2)
17	-	49.2 (C)
18	2.12 (m)	52.6 (CH)
19	1.31 1H (m)	38.5 (CH)
20	1.31 1H (m)	38.5 (CH)
21	1.43 1H (m)<br>1.31 1H (m)	30.2 (CH_2)
22	1.37 (1H, m), 0.91 (1H, m)	38.8(CH2)
23	3.51 1H (m), 3.20 1H (d, *J* = 10.5)	63.7 (t)
24	0.91 (3H, s)	15.0 (CH_3)
25	1.00 (3H, s)	18.3 (CH_3)
26	1.28 (3H, s)	18.2 (CH_3)
27	0.99 (3H, s)	18.3 (CH_3)
28	-	178.3 (C)
29	0.82 (3H, d, *J* = 6.4 Hz)	17.1 (CH_3)
30	0.91 (3H, d, J = 5.9 Hz)	21.1 (CH_3)

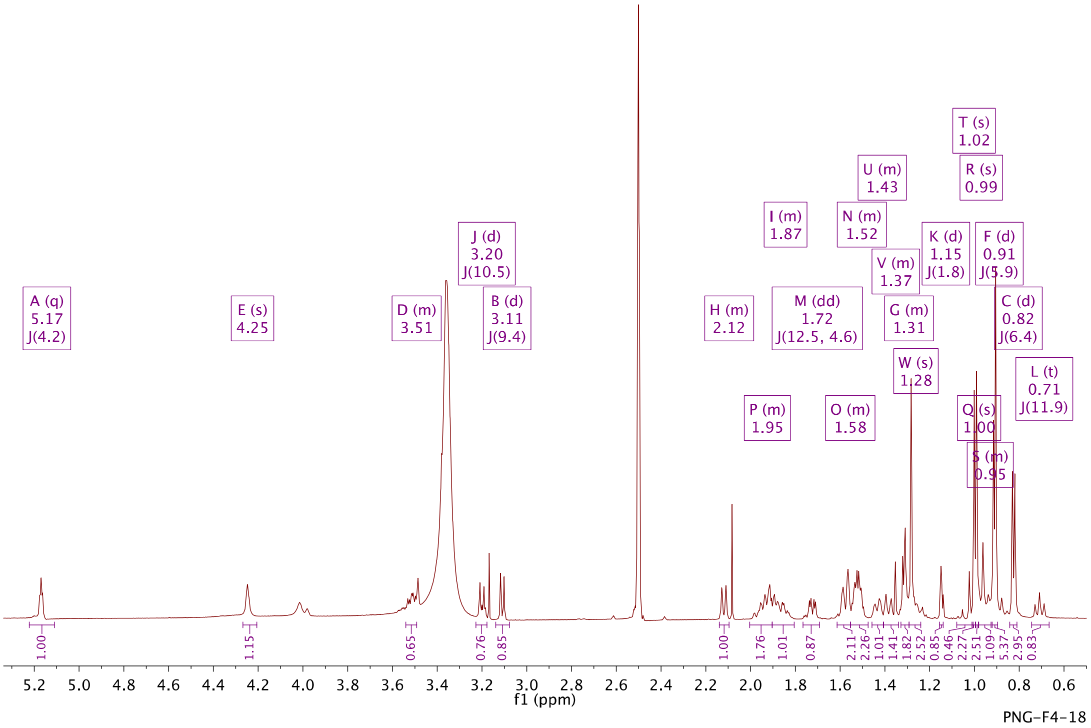

Figure S17 ¹H NMR (500 MHz) spectrum of madecassic acid in DMSO-d6.

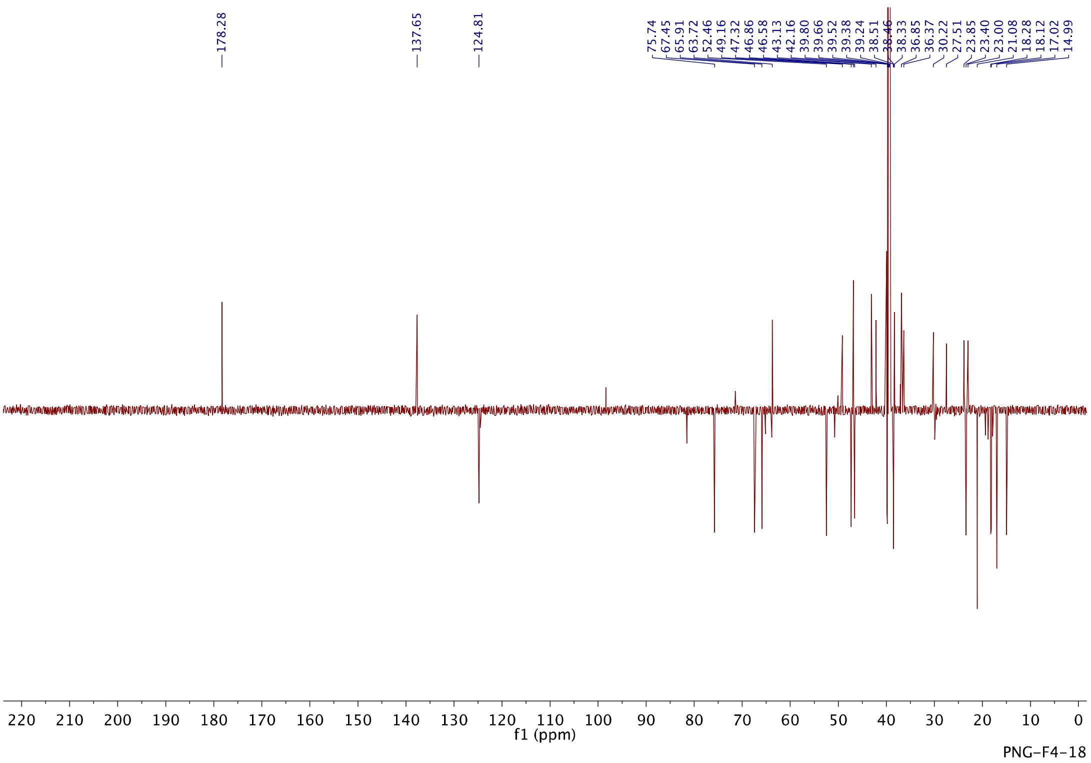

Figure S18 Dept q NMR (100 MHz) spectrum of madecassic acid in DMSO-d6.

S5 Characterization of PNG-F5 as 20-hydroxybetulin

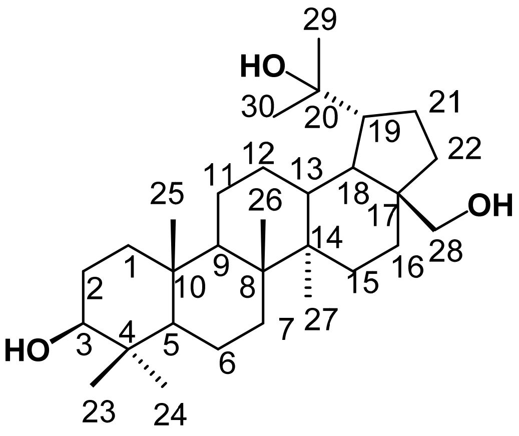

Figure S19 Structure of 20-hydroxybetulin

Table S5: ^{1}H (500MHz), ^{13}C (100MHz) data for 20-hydroxy betulin.

Position	Compound	
	**(9)**<br>**20-hydroxybetulin**	
	**^{1}H δ ppm (mult, *J*)**	**^{13}C δ ppm**
1	1.34 1H (m), 1.15 1H (m)	40.6 (CH_2)
2	1.77 1H (m), 1.68 1H (m)	27.8 (CH_2)
3	3.25 1H (dd, *J* = 11.10 5.53)	79.3 (CH)
4	-	38.2 (C)
5	0.87 1H (m)	55.9 (CH)
6	1.71 1H (m), 0.85 1H (m)	19.6 (CH_2)
7	2.34 1H (m), 1.65 1H (m)	35.2 (CH_2)
8	-	41.8 (C)
9	1.38 1H (m)	52.0 (CH)
10	-	39.1 (C)
11	1.81 1H (m), 1.05 1H (m)	29.1 (CH_2)
12	1.40 1H (m), 0.84 1H (m)	28.8 (CH_2)
13	1.40 1H (m)	36.0 (CH)
14	-	42.1 (C)
15	1.69 1H (m), 1.84 1H (m)	28.2 (CH_2)
16	1.91 1H (m), 1.30 1H (m)	30.1 (CH_2)
17	-	48.8 (C)
18	1.85 1H (m)	50.4 (CH)
19	1.37 1H (m)	48.4 (CH)
20	-	73.6 (C)
21	1.69 1H (m), 1.84 1H (m)	28.2 (CH_2)
22	2.30 1H (m), 0.88 1H (m)	33.8 (CH_2)
23	1.17 3H (s)	25.2 (CH_3)
24	0.793H (s)	16.1 (CH_3)
25	0.82 3H (s)	17.0 (CH_3)
26	1.53 3H (s)	18.4 (CH_3)
27	1.01 3H (s)	15.7 (CH_3)
28	3.93(d, *J* = 12.0), 4.02 (d, *J* = 12.0)	62.9 (CH_2)
29	1.77 3H (s)	24.5 (CH_3)
30	1.26 3H (s)	32.0 (CH_3)

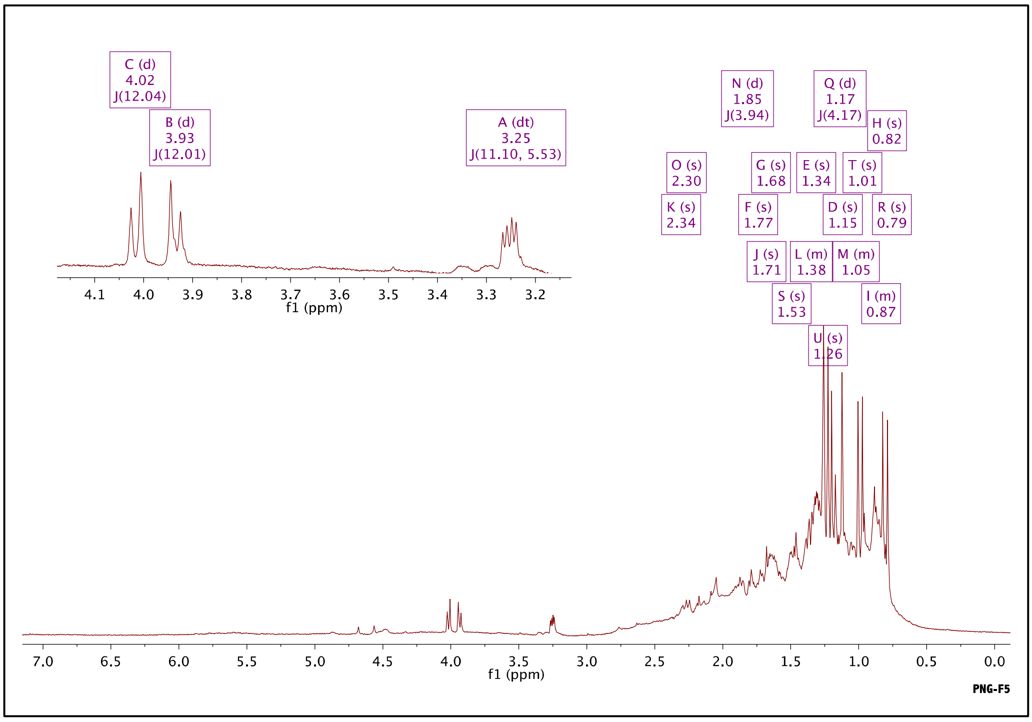

Figure S20 ^{1}H NMR (500 MHz) spectrum of 20-hydroxybetulin in $CDCl_3$

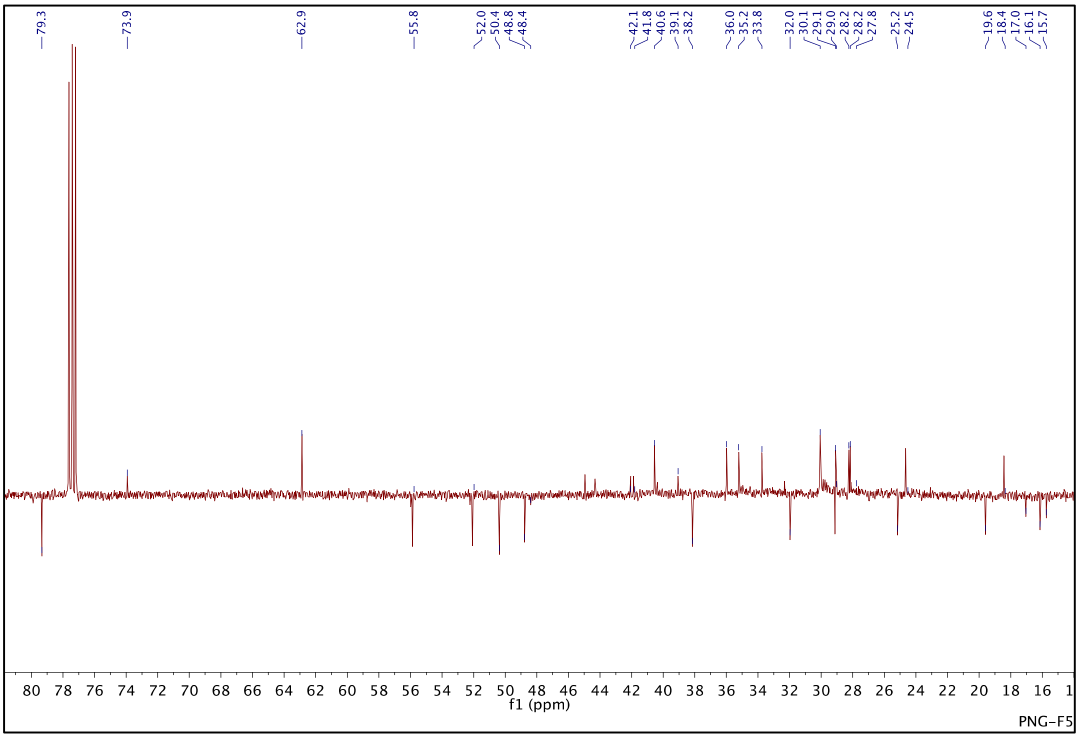

Figure S21 Dept q NMR (100 MHz) spectrum of 20-hydroxybetulin in $CDCl_3$

S6 Characterization of PNG-F6-S12 as Mangiferonic acid (Figure S27) and Ambonic acid (Figure S28)

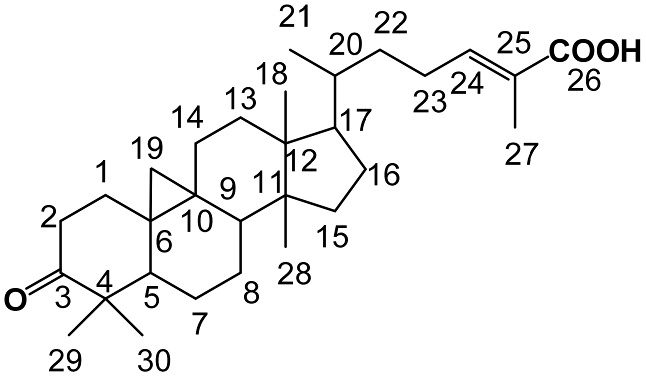

Figure S22 Structure of mangiferonic acid

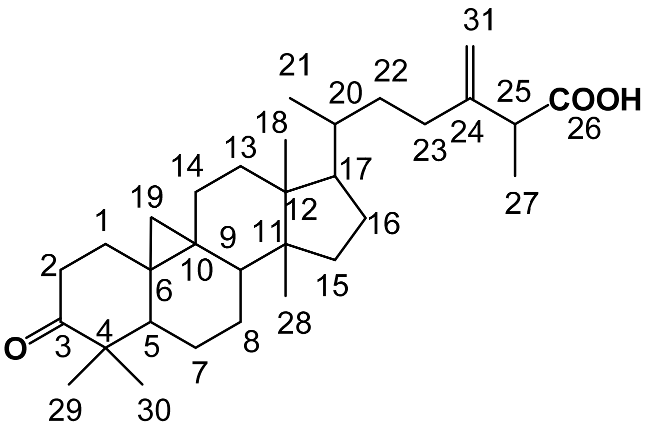

Figure S23 Structure of ambonic acid

Table S6: ^{1}H (500MHz), ^{13}C (100MHz) data for mangiferonic acid and ambonic acid.

**Position**	**Compound**			
**(1) Mangiferonic acid**			(5) Ambonic acid	
	^{1}H δ ppm (mult, J)	^{13}C δ ppm	^{1}H δ ppm (mult, J)	^{13}C δ ppm
1	1.88 1H (m), 1.57 1H (m)	33.4 (CH_2)	1.68 1H (m), 1.40 1H (m)	32.7 (CH_2)
2	2.73 1H (td, 13.84, 6.40)<br>2.33 1H (td, 14.17, 3.49)	37.4 (CH_2)	2.73 1H (td, 13.84, 6.40)<br>2.33 1H (td, 14.17, 3.49)	37.4 (CH_2)
3	-	216.6 (C)	-	216.6 (C)
4	-	50.2 (C)	-	50.2 (C)
5	1.73 1H (dd, 12.17, 4.33)	48.4 (CH)	1.73 1H (dd, 12.17, 4.33)	48.4 (CH)
6	1.57 1H (m), 0.97 1H (m)	21.5 (CH_2)	1.57 1H (m), 0.97 1H (m)	21.4 (CH_2)
7	1.40 1H (m), 1.16 1H (m)	25.9 (CH_2)	1.40 1H (m), 1.16, 1H (m)	25.9 (CH_2)
8	1.58 1H (m)	47.8 (CH)	1.58 1H (m)	47.8 (CH)
9	-	21.0 (C)	-	21.0 (C)
10	-	25.9 (C)	-	25.9 (C)
11	2.07 1H (m), 1.19 (m)	26.7 (CH_2)	2.07 1H (m), 1.19 1H (m)	26.6 (CH_2)
12	1.68 1H (m), 1.19 1H (m)	32.7 (CH_2)	2.07 1H (m), 1.68 1H (m)	32.7 (CH_2)
13	-	45.3 (C)	-	45.3 (C)
14	-	48.7 (C)	-	48.7 (C)
15	1.58 1H (m), 1.40 1H (m)	35.5 (CH_2)	1.58 1H (m), 1.40 1H(m)	35.5 (CH_2)
16	1.91 1H (m), 1.68 1H (m)	28.0 (CH_2)	1.91 1H (m), 1.68 1H (m)	28.0 (CH_2)
17	1.68 1H (m)	52.2,1H (CH)	1.68, 1H (m)	52.2 (CH)
18	1.01 3H (s)	18.0 (CH_3)	01.02 3H (s)	18.0 (CH_3)
19	0.81 1H (d, 3.55)<br>0.59 1H (d, 3.73)	29.5 (CH_2)	0.81 1H (d, 3.55)<br>0.59 1H (d, 3.73)	29.5 (CH_2)
20	1.40 1H (m)	35.9 (CH)	1.40 1H (m)	35.9 (CH)
21	0.94 3H (d, 6.31)	18.2 (CH_3)	0.94 3H (d, 6.31)	18.2 (CH_3)
22	1.19 1H (m), 1.58 1H (m)	34.7 (CH_2)	1.19 1H (m), 1.58 1H (m)	34.5 (CH_2)
23	2.28 1H (m), 2.15 1H (m)	25.8 (CH_2)	2.21 2H (m)	31.6 (CH_2)
24	6.93 1H (t, 7.52)	145.8 (CH)	-	148.0 (C)
25	-	126.6 (C)	3.19 1H (q, 7.08)	45.6 (CH)
26	-	173.3 (C)	-	180.2 (C)
27	1.86 3H (s)	11.9 (CH_3)	1.33 3H (d, 7.0)	16.3 (CH_3)
28	1.07 3H (s)	22.1 (CH_3)	1.07 3H (s)	19.3 (CH_3)
29	1.12 3H (s)	21.1 (CH_3)	1.12 3H (s)	22.1 (CH_3)
30	0.93 3H (s)	19.3 (CH_3)	0.913H (s)	20.7 (CH_3)
31	-	-	4.99 (1H, s), 4.95 (1H, s)	111.0 (CH_2)

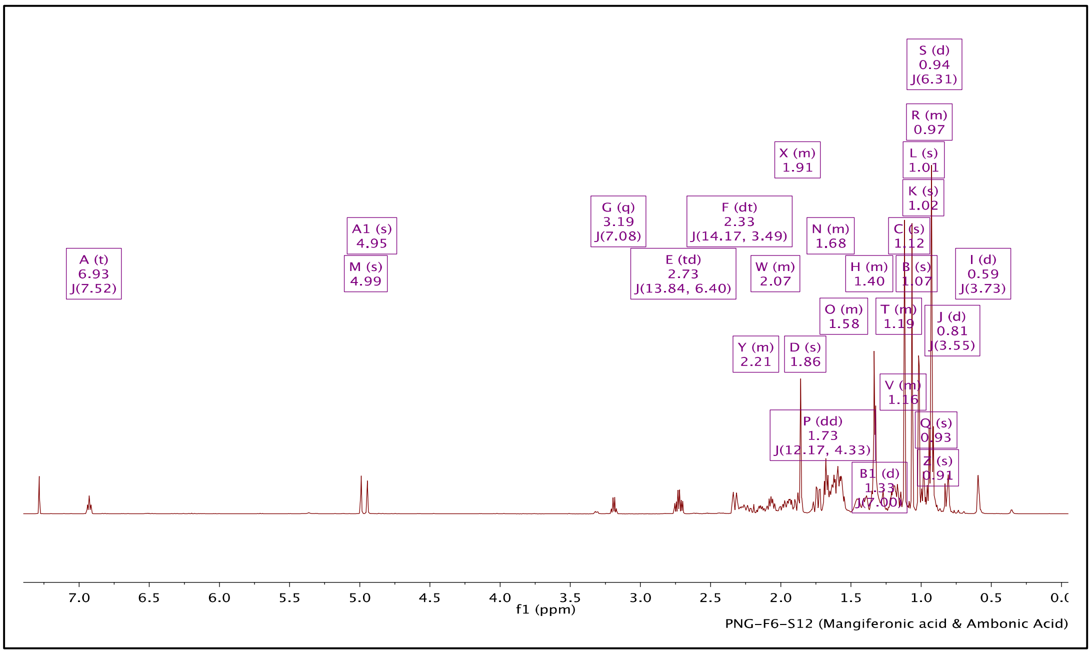

Figure S24 ^{1}H NMR (500 MHz) spectrum of the mixture of mangiferonic acid and ambonic acid in $CDCl_3$

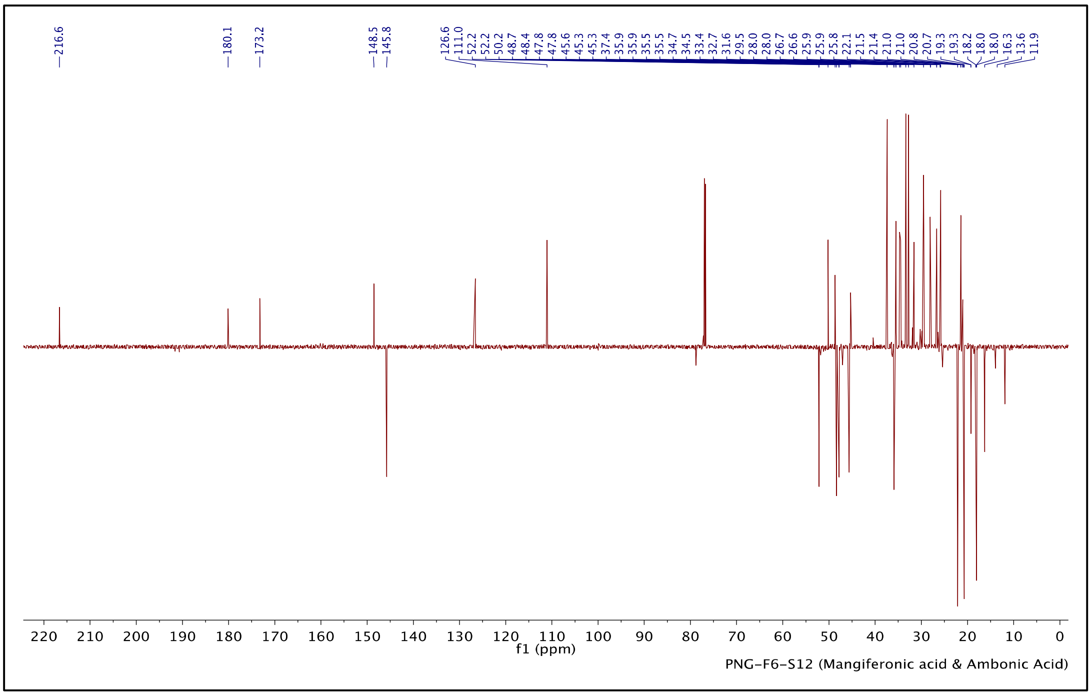

Figure S25 Dept q NMR (100 MHz) spectrum of the mixture of mangiferonic acid and ambonic acid in $CDCl_3$

S7 Characterization of PNG-F6-S15 as Isomangiferolic acid (Figure S32 and Ambolic acid (Figure S33)

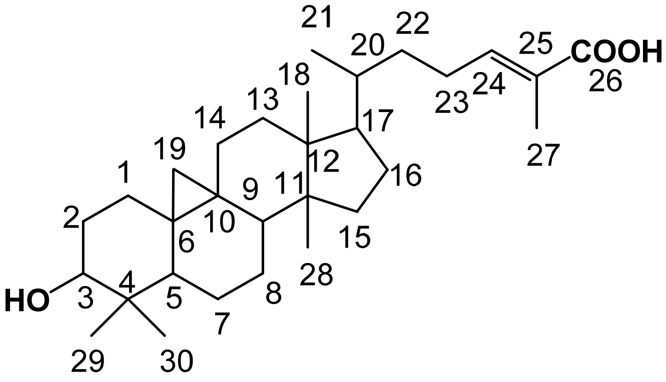

Figure S26 Structure of Isomangiferolic acid

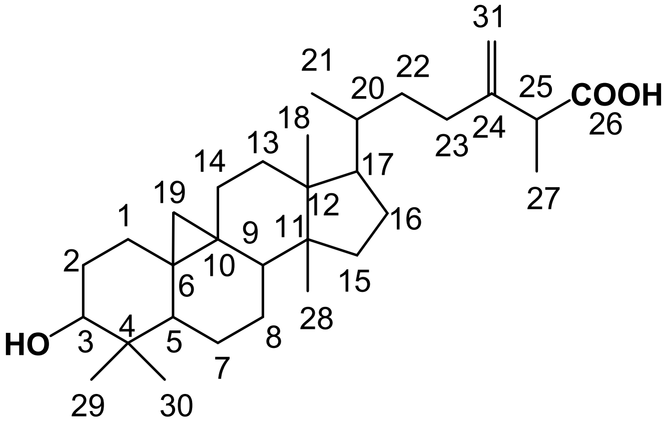

Figure S27 Structure of Ambolic acid

Table S7: ^{1}H (500MHz), ^{13}C (100MHz) data for isomangiferolic acid and ambolic acid.

Position	Compound			
	**(2) Isomangiferolic acid**		**(6) Ambolic acid**	
	**^{1}H δ ppm (mult, *J*)**	**^{13}C δ ppm**	**^{1}H δ ppm (mult, *J*)**	**^{13}C δ ppm**
1	1.74 1H (m), 1.04 1H (m)	27.5 (CH_2)	1.92 1H (m), 1.27 1H (m)	32.0 (CH_2)
2	1.92 1H (m), 1.64 1H (m)	28.5 (CH_2)	1.74 1H (m), 1.52 1H (m)	30.3 (CH_2)
3	3.5 1H (m)	77.1 (CH)	3.5 1H (m)	77.1 (CH)
4	-	39.5 (C)	-	40.5 (C)
5	1.831H (m)	41.1 (CH)	1.27 1H (m)	47.1 (CH)
6	1.65 1H (m), 0.80 1H (m)	21.1 (CH_2)	1.651H (m), 0.80 1H (m)	21.1 (CH_2)
7	1.27 1H (m), 1.14 1H (m)	25.7 (CH_2)	1.27 1H (m), 1.14 1H (m)	26.0 (CH_2)
8	1.52 1H (m)	48.0 (CH)	1521H (m)	48.1 (CH)
9	-	19.8 (C)	-	19.8 (C)
10	-	26.5 (C)	-	26.5 (C)
11	2.03 1H (m), 1.14 (m)	26.3 (CH_2)	2.03 1H (m), 1.14 1H (m)	26.3 (CH_2)
12	1.65 2H (m)	32.9 (CH_2)	1.65 2H (m)	32.9 (CH_2)
13	-	45.3 (C)	-	45.3 (C)
14	-	48.9 (C)	-	48.9 (C)
15	1.34 2H (m)	35.5 (CH_2)	1.34 2H (m)	35.5 (CH_2)
16	1.92 1H (m), 1.30 1H (m)	28.1 (CH_2)	1.92 1H (m), 1.30 1H (m)	28.1 (CH_2)
17	1.65 1H (m)	52.2 (CH)	1.65 1H (m)	52.2 (CH)
18	0.90 3H (s)	18.0 (CH_3)	0.90 3H (s)	18.1 (CH_3)
19	0.54 1H (d, 4.09)<br>0.37 1H (d, 4.03)	29.8 (CH_2)	0.54 1H (d, 4.09)<br>0.37 1H (d, 4.03)	29.8 (CH_2)
20	1.44 1H (m)	36.0 (CH)	1.44 1H (m)	36.0 (CH)
21	0.92 3H (d, 6.31)	18.0 (CH_3)	0.94 3H (d, 6.31)	18.1 (CH_3)
22	1.58 1H (m), 1.19 1H (m)	34.8 (CH_2)	1.58 1H (m), 1.19 1H (m)	34.6 (CH_2)
23	2.27 1H (m), 2.14 1H (m)	25.9 (CH_2)	2.03 1H (m), 1.92 1H (m)	25.9 (CH_2)
24	6.92 1H (t d, 7.52, 1.57)	145.7 (CH)	-	148.7 (C)
25	-	126.6 (C)	3.19 1H (q, 7.13)	45.6 (CH)
26	-	173.0 (C)	-	45.7 (C)
27	1.86 3H (s)	12.0 (CH_3)	1.32 3H , 7.05)	16.3 (CH_3)
28	0.99 3H (s)	25.8 (CH_3)	0.99 3H (s)	25.4(CH_3)
29	0.97 3H (s)	21.2 (CH_3)	0.83 3H (s)	14.0 (CH_3)
30	0.92 3H (s)	19.3 (CH_3)	0.92 3H (s)	19.3 (CH_3)
31	-	-	4.98 (1H, s)<br>4.94 (1H, s)	111.0 (CH_2)

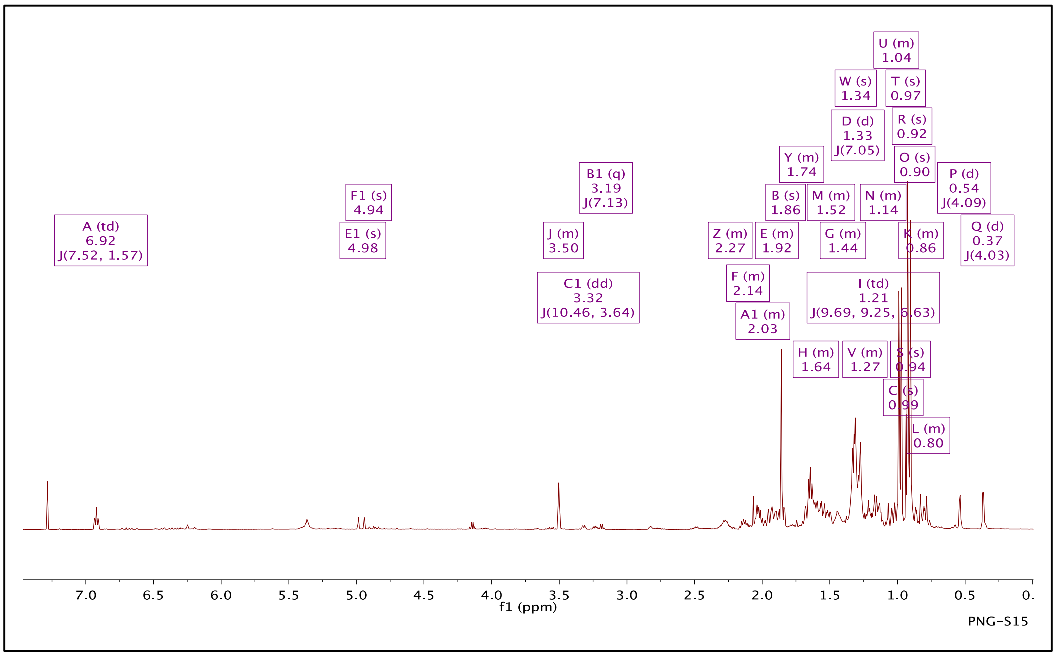

Figure S28 ^{1}H NMR (500 MHz) spectrum of the mixture of isomangiferolic acid and ambolic acid in $CDCl_3$.

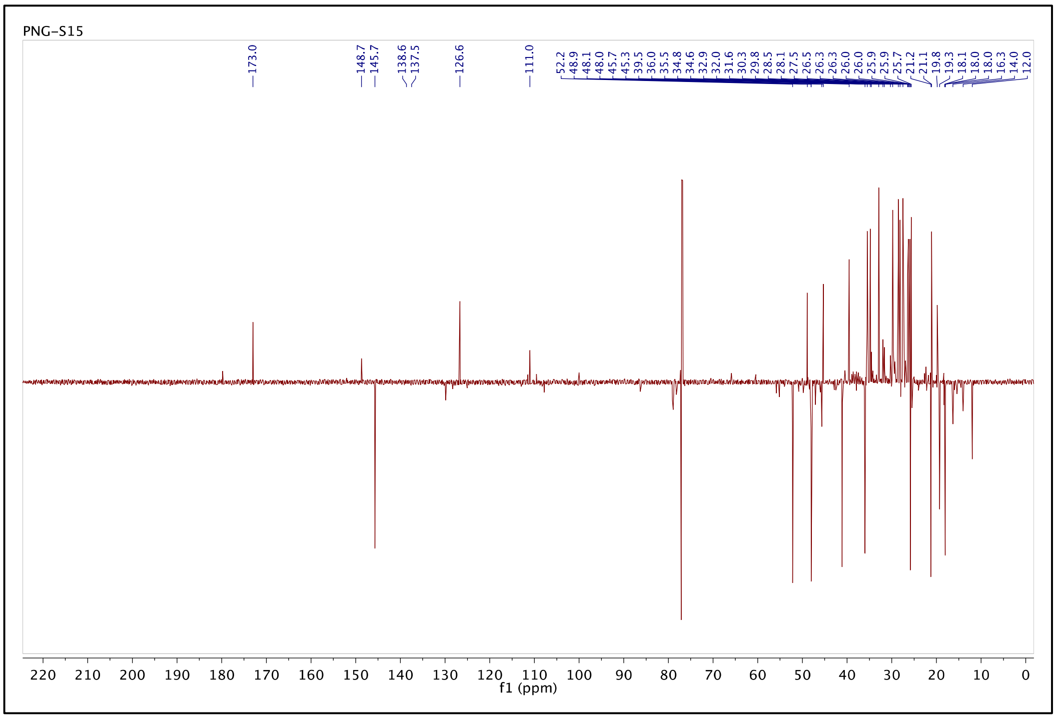

Figure S29 Dept q NMR (100 MHz) spectrum of the mixture of isomangiferolic acid and ambolic acid in $CDCl_3$.

molecules MDPI

S8 Characterisation of PNGS17 as hydroxymangiferolic acid

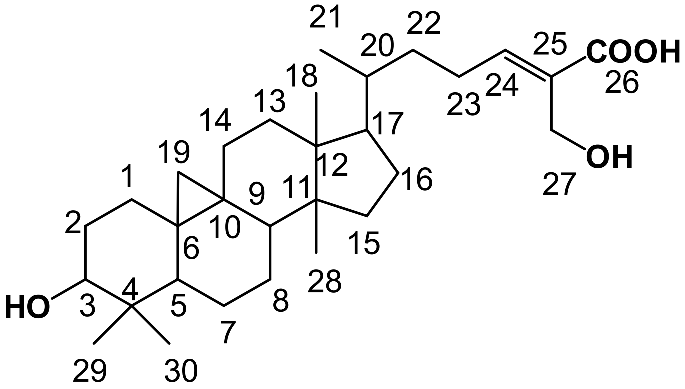

Figure S30 Structure of 27- Hydroxyisomangiferolic acid

Table S8 ^{1}H (500MHz), ^{13}C (100MHz) data for 27- Hydroxyisomangiferolic acid.

Position	Compound	
	**(3)**<br>**27- Hydroxyisomangiferolic acid**	
	**^{1}H δ ppm (mult, *J*)**	**^{13}C δ ppm**
1	1.851H (m), 1.26 1H (m)	28.1 (CH_2)
2	1.92 1H (m), 1.55 1H (m)	29.1 (CH_2)
3	3.06 1H (q, 4.75)	77.1 (CH)
4	-	39.6 (C)
5	1.78 1H (m)	40.6 (CH)
6	1.47 1H (m), 0.75 1H (m)	21.1 (CH_2)
7	1.26 1H (m), 1.14 1H (m)	25.0 (CH_2)
8	1.55 1H (m)	47.8 (CH)
9	-	19.5 (C)
10	-	26.7 (C)
11	2.21 1H (s), 1.14 1H (m)	26.3 (CH_2)
12	1.55 2H (m)	33.0 (CH_2)
13	-	45.3 (C)
14	-	48.9 (C)
15	1.26 2H (m)	35.5 (CH_2)
16	1.92 1H (m), 1.26 1H (m)	28.2 (CH_2)
17	1.60 1H (m)	52.1 (CH)
18	0.94 3H (s)	18.3 (CH_3)
19	0.49 1H (d, 3.96), 0.30 1H (d, 3.86)	29.7 (CH_2)
20	1.47 1H (m)	35.9 (CH)
21	0.87 3H (d, 3.55)	18.4 (CH_3)
22	1.55 1H (m), 1.14 1H (m)	35.5 (CH_2)
23	2.29 1H (m), 2.12 1H (m)	26.1 (CH_2)
24	6.62 1H (t, 7.7)	145.8 (CH)
25	-	132.8 (C)
26	-	171.1 (C)
27	4.11 2H (s)	55.8 (CH_2)
28	0.94 3H (s)	26.1 (CH_3)
29	0.88 3H (s)	21.7 (CH_3)
30	0.88 3H (s)	19.8 (CH_3)

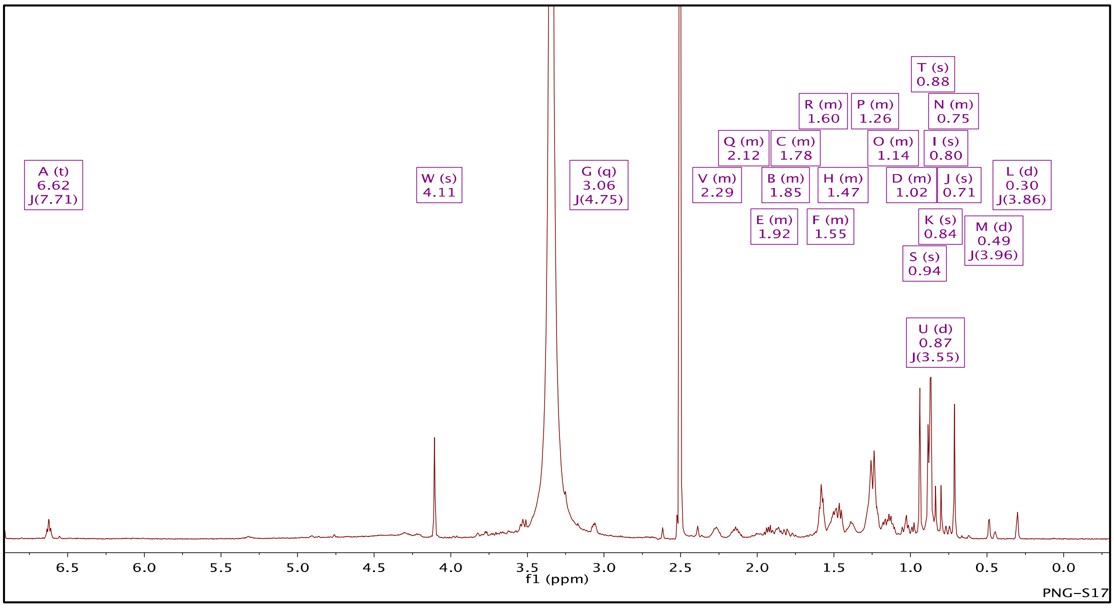

Figure S31 ^{1}H NMR (500 MHz) spectrum for 27- hydroxyisomangiferolic acid in DMSO-d6

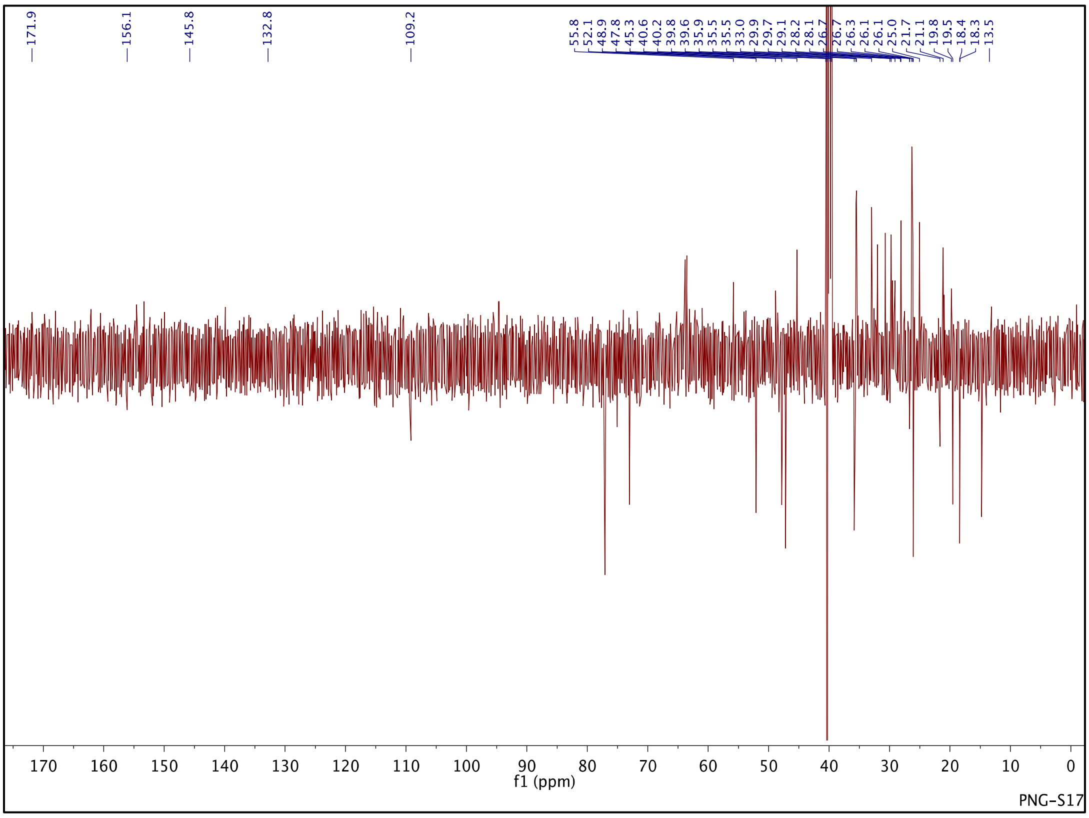

Figure S32 Dept q (100 MHz) spectrum of 27- hydroxyisomangiferolic acid in DMSO-d6**.**
